# Effect of 2 Integrated Interventions on Alcohol Abstinence and Viral Suppression Among Vietnamese Adults With Hazardous Alcohol Use and HIV: A Randomized Clinical Trial

**DOI:** 10.1001/jamanetworkopen.2020.17115

**Published:** 2020-09-18

**Authors:** Vivian F. Go, Heidi E. Hutton, Tran V. Ha, Geetanjali Chander, Carl A. Latkin, Nguyen V. T. Mai, Bui X. Quynh, Vu Nguyen, Teerada Sripaipan, Kathryn E. Lancaster, Natalie Blackburn, Rebecca B. Hershow, David W. Dowdy, Constantine Frangakis

**Affiliations:** 1 Department of Health Behavior, Gillings School of Global Public Health, University of North Carolina at Chapel Hill; 2 Department of Psychiatry and Behavioral Sciences, School of Medicine, Johns Hopkins University, Baltimore, Maryland; 3 University of North Carolina Vietnam, Hanoi, Vietnam; 4 Division of General Internal Medicine, Department of Medicine, Johns Hopkins University, Baltimore, Maryland; 5 Department of Epidemiology, Bloomberg School of Public Health, Johns Hopkins University, Baltimore, Maryland; 6 Department of Health, Behavior, and Society, Bloomberg School of Public Health, Johns Hopkins University, Baltimore, Maryland; 7 Department of Medicine, Johns Hopkins University, Baltimore, Maryland; 8 Vinmec Healthcare System, Hanoi, Vietnam; 9 Division of Epidemiology, College of Public Health, The Ohio State University, Columbus, Ohio; 10 Division of Infectious Diseases, Department of Epidemiology, Bloomberg School of Public Health, Johns Hopkins University, Baltimore, Maryland; 11 Division of Global Disease Epidemiology and Control, Department of International Health, Bloomberg School of Public Health, Johns Hopkins University, Baltimore, Maryland; 12 Department of Biostatistics, Bloomberg School of Public Health, Johns Hopkins University, Baltimore, Maryland

## Abstract

**Importance:**

Hazardous and heavy alcohol use is common among people living with HIV and may decrease antiretroviral therapy (ART) adherence, but limited data exist from randomized clinical trials about the effects of interventions on viral load.

**Objective:**

To compare the efficacy of 2 scalable ART clinic–based interventions on alcohol use and viral suppression.

**Design, Setting, and Participants:**

This 3-group randomized clinical trial was conducted among 440 adults with HIV who were being treated at 7 ART clinics in Thai Nguyen, Vietnam. Adults receiving ART with hazardous alcohol use (Alcohol Use Disorders Identification Test–Consumption score ≥4 for men or ≥3 for women) and no plans to leave Thai Nguyen were included. Data were collected from March 2016 to May 2018 and analyzed from June 2018 to February 2020.

**Interventions:**

Participants were randomly assigned (1:1:1) to standard of care (SOC), a combined intervention of motivational enhancement therapy and cognitive behavioral therapy (6 in-person sessions of 1 hour each and 3 optional group sessions), or a brief intervention with similar components as the combined intervention but consisting of 2 shorter in-person sessions and 2 telephone sessions.

**Main Outcomes and Measures:**

The primary study outcomes were percentage of days abstinent from alcohol, confirmed using the alcohol biomarker phosphatidylethanol, and viral suppression at 12 months after enrollment.

**Results:**

A total of 440 eligible individuals (mean [SD] age, 40.2 [5.8] years; 426 [96.8%] men) were enrolled; 147 (33.4%) were assigned to the combined intervention, 147 (33.4%) to the brief intervention, and 146 (33.2%) to SOC. In the combined intervention group, 112 participants (76.2%) attended all 6 sessions, and in the brief intervention group, 124 (84.4%) attended all 4 sessions; in the whole sample, 390 (88.6%) completed 12 months of follow-up. At 12 months, the mean (SE) percentage of days abstinent was 65% (3.1%) among those in the combined intervention group, 65% (3.2%) among those in the brief intervention group, and 50% (3.4%) among those in the in the SOC group (Cohen *d* for combined intervention vs SOC and brief intervention vs SOC: 39%; 95% CI, 15% to 64%). Viral suppression (ie, <20 copies of HIV-1 RNA per milliliter) at 12 months was higher after the brief intervention than SOC (difference, 11%; 95% CI, 2% to 20%), but the difference between the combined intervention and SOC was not significantly different (difference, 5%; 95%, CI, –5% to 15%).

**Conclusions and Relevance:**

In this study, the brief intervention resulted in a significant increase in percentage of days abstinent from alcohol and a significant increase in viral suppression after 12 months. Future implementation science studies evaluating scale-up of the brief intervention are needed.

**Trial Registration:**

ClinicalTrials.gov Identifier: NCT02720237

## Key Points

**Question:**

What is the effect of 2 clinic-based behavioral interventions of varying intensity on alcohol use and viral suppression among individuals with HIV and hazardous alcohol use?

**Findings:**

In this 3-group randomized clinical trial involving 440 participants, the combined intervention and brief intervention, which both incorporated motivational enhancement therapy and cognitive behavioral therapy, significantly increased the percentage of days abstinent from alcohol at 12 months compared with the standard of care. Viral suppression at 12 months was significantly higher after the brief intervention than standard of care but not significantly higher after the combined intervention than standard of care.

**Meaning:**

These findings support the use of the brief intervention in antiretroviral therapy clinics to reduce alcohol use and increase viral suppression in this high-risk population.

## Teaser

This randomized clinical trial compares the efficacy of 2 scalable antiretroviral clinic–based interventions on alcohol use and viral suppression among adults with hazardous alcohol use and HIV in Vietnam.

## Introduction

Hazardous alcohol use is highly prevalent among people living with HIV (PWH).^1^ In a large multisite clinical cohort of PWH in the United States, 27% reported hazardous alcohol use and 34% reported binge drinking.^2^ In low-income and middle-income countries, hazardous alcohol use among PWH is common, with 20% to 46% of PWH reporting hazardous alcohol use.^3,4,5,6,7,8,9^ Alcohol use has been associated with decreased adherence to antiretroviral therapy (ART)^10,11,12^ and decreased viral suppression.^13^ Alcohol use is also associated with elevated sexual and injection risk behaviors that increase the likelihood of HIV transmission.^7,14,15,16^ Despite the known adverse health consequences of alcohol use, there is a lack of evidence-based interventions for PWH,^17,18,19,20,21^ and hazardous and heavy alcohol consumption are frequently not addressed in HIV health care settings.

Hazardous and heavy alcohol use in specific subpopulations of PWH, including people who inject drugs (PWID), may place them at even higher risk for health consequences due to comorbidities as well as transmission of HIV and other infections. Hazardous alcohol use among PWID who are living with HIV is independently associated with decreased ART adherence and viral suppression^13^ as well as sharing needles and/or syringes, having multiple sex partners, and engaging in sex without condoms.^10,15,22^

We conducted a 3-group randomized clinical trial to compare the effectiveness of the combined intervention and the brief intervention integrated into ART clinics with the standard of care (SOC) on percentage of days abstinent from alcohol (assessed with timeline follow-back interviews and confirmed using the alcohol biomarker phosphatidylethanol)^23^ and viral suppression at 12 months after enrollment. The combined intervention and brief intervention have been highly effective in reducing alcohol use among people living with HIV with hazardous or heavy alcohol use in US settings^24,25,26^ and were selected for their potential applicability to the Vietnamese context.^27^

## Methods

### Study Setting

The study was conducted in Thai Nguyen, a semi-urban province in North Vietnam located 75 kilometers north of Hanoi with a population of approximately 1 million residents. Thai Nguyen has 12 government outpatient ART clinics that are the sole source of ART medications in the province. We recruited participants and conducted the study in the 7 largest outpatient ART clinics.

### Ethical Approvals

The study was approved by the institutional review boards at the University of North Carolina Gillings School of Global Public Health, the Johns Hopkins University Bloomberg School of Public Health, and the Thai Nguyen Center for Preventive Medicine. All study participants provided written informed consent in Vietnamese. The study followed the Consolidated Standards of Reporting Trials (CONSORT) reporting guideline. The trial protocol and statistical analysis plan are available in Supplement 1.

### Randomization and Masking

Participants were randomly assigned to the combined intervention, the brief intervention, or the SOC group at a ratio of 1:1:1. To achieve this, a randomization schedule was generated in SAS version 9.4 (SAS Institute) using permuted-block randomization with a block size of 3, such that each block contained a random assignment to each of the 3 groups. Participants randomly assigned within 1 triplet block all belonged to the same ART clinic. Masking in the field was not feasible due to the nature of the intervention; however, analysts and principal investigators were masked to study assignment until the analyses for this paper were complete.

### Eligibility Criteria

Eligibility criteria included being enrolled at a study ART clinic and receiving ART; having hazardous alcohol use, defined as having an Alcohol Use Disorders Identification Test–Consumption score of at least 4 for men or at least 3 for women; being aged 18 years or older; and planning on residing in Thai Nguyen for the next 24 months. The sole exclusion criterion was having a Clinical Institute Withdrawal Assessment score of at least 10, out of concerns for risk of alcohol withdrawal.

### Recruitment Procedures

Recruitment began in March 2016 and continued through May 2017. Participants were followed up for 12 months. Any ART client who accessed care in the study clinics during the recruitment period was approached for participation (eTable 1 in Supplement 2). Once saturation was reached (ie, the study team did not identify any new potential participants for at least 1 week), we moved to recruitment in the next clinic, using a random ordering of clinics. HIV clinicians introduced the study to patients and referred them to study staff if interested, at which time eligibility was assessed and written informed consent was obtained. Viral load was not assessed prior to enrollment.

### Study Procedures

Participants in all 3 groups received a standard Ministry of Health recommendation from their HIV clinician to decrease alcohol use and a referral to harm reduction services and to treatment of hepatitis B and C viruses, sexually transmitted diseases, and tuberculosis. The processes for selecting and culturally adapting the 2 interventions are described in detail elsewhere.^27^ We conducted training and provided supervision through weekly meetings using a completion checklist and the Yale Adherence and Competence Scale (YACS; range 1-7, with 7 indicating excellent fidelity)^28^ to ensure sessions were standardized across our team of paraprofessional counselors who delivered the interventions. A team of 2 counselors, 1 woman and 1 man, was assigned to the combined intervention group, and a different counseling team, 1 woman and 1 man, was assigned to the brief intervention group.

Because the concept of alcohol reduction is novel in this setting, we selected a combined intervention approach that includes cognitive behavioral therapy and motivational enhancement therapy.^27,29^ The brief intervention has similar components as the combined intervention but is briefer.

The combined group included 6 individual face-to-face sessions delivered 1 week apart and 3 optional group sessions delivered concurrently. The first session used motivational enhancement therapy, adopting a guiding but nonjudgmental style to enhance motivation. Subsequent sessions used cognitive behavioral therapy, building skills to effectively refuse alcohol, to manage cravings and high-risk situations for alcohol use, and to develop self-efficacy. Sessions also provided information on the harmful effects of drinking on HIV and overall health. Group sessions reinforced skills learned in the individual sessions and provided a forum to discuss experiences (eTable 2 in Supplement 2).

Participants in the brief intervention group participated in 2 individual face-to-face sessions and 2 individual booster telephone sessions. Based on Project TrEAT,^30^ the content of the brief intervention sessions included elements used in the combined intervention, including information about alcohol and its effects as well as alcohol behavior change strategies (eTable 2 in Supplement 2). Face-to-face sessions occurred approximately 1 month apart, and telephone sessions occurred 2 to 3 weeks after each face-to-face session.

### Data Collection

In a private room at the ART clinic, participants completed study visits at enrollment and at 3, 6, and 12 months after enrollment. At the end of each visit, participants were given 100 000 Vietnamese dong (approximately US $4.30) to compensate them for their lost work time; they were also reimbursed for their expenses for travel to and from the study site. Behavioral measures, including ART adherence, were based on self-report. Depression and anxiety were assessed using the Patient Health Questionnaire–9 and Generalized Anxiety Disorder–7 scores, respectively, and alcohol dependence or alcohol abuse was defined using the Mini-International Neuropsychiatric Interview version 5.0.0. After the questionnaires were completed, interviewers administered the timeline follow-back interview.^31^ Participants provided blood samples at every visit except screening for the following tests: CD4^+^ T-cell count, HIV viral load (HIV-1 RNA test, COBAS AmpliPrep and COBAS TaqMan HIV-1 Test, Roche Molecular Systems), and hepatitis B surface antigen rapid test (Alere Determine hepatitis B surface antigen reagent). Dried blood spots were collected and shipped to US Drug Testing Laboratories for phosphatidylethanol testing to detect any alcohol use during the 3 weeks before the study visit. Phosphatidylethanol is a direct metabolite of alcohol consumption that serves as a biomarker for alcohol consumption during the preceding 3 weeks.^23^ Phosphatidylethanol levels of less than 10 ng/mL have demonstrated a sensitivity of 88% and a specificity of 88.5% with an area under the curve operating characteristic curve of 0.92 for detection of any recent drinking (past 21 days).^23^

### Outcomes

The primary end points were the effect of each intervention, compared with the standard of care, on self-reported percentage of days abstinent from alcohol measured by the timeline follow-back interview and viral suppression at 12 months after enrollment. Viral load suppression was defined as less than 20 copies of HIV-1 RNA per milliliter.

Key secondary outcomes were number of drinks per drinking day and number of heavy drinking days measured by the timeline follow-back interview. A heavy drinking day was defined as more than 4 drinks per day for men and more than 3 drinks per day for women.^32^ We assessed the association between phosphatidylethanol level of less than 8 ng/mL, the established threshold for detecting alcohol abstinence, and self-reported abstinence during the past 3 weeks at baseline, 3-month follow-up, and 12-month follow-up.

### Statistical Analysis

The study was designed to have 80% power to detect the following: (1) greater than 50% effect size in percentage of abstinent days at 12-month follow-up minus baseline, comparing the combined intervention vs SOC (or the brief intervention vs SOC); (2) greater than 35% effect size in percentage abstinent days at 12-month follow-up minus baseline, comparing the combined intervention vs the brief intervention; and (3) greater than 6% change in viral suppression at 12-month follow-up minus baseline, comparing the combined intervention vs SOC (or the brief intervention vs SOC). For each outcome, we evaluated the extent to which the mean values differed across groups after baseline by intention-to-treat. To do this, first we estimated the proportion of patients in each of the 3 groups for whom the outcome was measured and compared among groups using the deviance χ^2^ test. We also estimated the mean outcome level in each group and compared among groups using a Wald χ^2^ test. We also assessed the possibility of selectively differential attrition across arms at each postbaseline visit. To do this, we estimated and compared the mean baseline outcome among groups for the patients who provided outcome data at that postbaseline visit. If an imbalance was found, we also produced postbaseline regression-adjusted outcome estimates by recalibrating the baseline distribution of that group to the baseline distribution across all groups combined.^33^ In short, the overall group distribution of the baseline outcome can be estimated at each value *x* as 1 / total sample size *n*. Therefore, if the regression of the mean postbaseline outcome *y* given baseline value *x* within arm *a* is estimated as ȳ_a_ × x, then the regression-adjusted outcome average in that arm is (1 / n) × Σ_x_ × (ȳ_a_ × x).

Statistical analyses were conducted in R version 3.4.3 (R Project for statistical computing) and SAS version 9.4 (SAS Institute). Statistical significance was set at *P* < .05, and all tests were 2-tailed.

## Results

During 15 months, 1559 screening evaluations were performed, and 440 PWH with hazardous or heavy drinking (mean [SD] age, 40.2 [5.8] years; 426 [96.8%] men) were enrolled (Table 1). Based on the 1:1:1 random allocation, 147 (33.4%) were assigned to the combined intervention group, 147 (33.4%) were assigned to the brief intervention group, and 146 (33.2%) were assigned to the SOC group (Figure). During follow-up, 15 participants (3.4%) died (6 [40.0%] in the brief intervention group, 6 [40.0%] in the standard of care group, and 3 [20.0%] in the combined intervention group). Excluding participants who died, 405 of 435 participants (93.1%) completed the 3-month follow-up, 410 of 432 (94.9%) completed the 6-month follow-up, and 390 of 425 (91.8%) completed the 12-month follow-up. The most common reasons for missed visits at 12 months were incarceration and relocation. By the 12-month visit, 390 of 440 participants (88.6%) provided abstinence and viral load outcomes.

**Table 1.** Baseline Characteristics by Group

Characteristic	No. (%)			
	Overall (N = 440)	Combined intervention (n = 147)	Brief intervention (n = 147)	Standard of care (n = 146)
AUDIT score, median (IQR)	12 (9-16)	12 (9-16)	12 (9-16)	11 (9-15)
Currently has alcohol dependence or alcohol abuse^a^				
No	256 (58.2)	92 (62.6)	76 (51.7)	88 (60.3)
Yes	184 (41.8)	55 (37.4)	71 (48.3)	58 (39.7)
Age, mean (SD), y	40.2 (5.8)	40.4 (5.8)	39.8 (5.6)	40.3 (5.9)
Sex				
Men	426 (96.8)	145 (98.6)	140 (95.2)	141 (96.6)
Women	14 (3.2)	2 (1.4)	7 (4.8)	5 (3.4)
Education				
No high school	312 (70.9)	106 (72.1)	100 (68)	106 (72.6)
Any high school or more	128 (29.1)	41 (27.9)	47 (32)	40 (27.4)
Marital status				
Single	66 (15)	20 (13.6)	23 (15.6)	23 (15.8)
Married or living with a partner	318 (72.3)	108 (73.5)	105 (71.4)	105 (71.9)
Widowed, divorced, or separated	56 (12.7)	19 (12.9)	19 (12.9)	18 (12.3)
Employment status				
<Full-time	202 (45.9)	65 (44.2)	69 (46.9)	68 (46.6)
Full-time	238 (54.1)	82 (55.8)	78 (53.1)	78 (53.4)
Spent the night outside in the past 3 mo				
No	434 (98.6)	147 (100)	142 (96.6)	145 (99.3)
Yes	6 (1.4)	0	5 (3.4)	1 (0.7)
Ever injected drugs in lifetime				
No	84 (19.1)	27 (18.4)	25 (17.0)	32 (21.9)
Yes	356 (80.9)	120 (81.6)	122 (83.0)	114 (78.1)
Any injecting drug use in the past 3 mo^b^				
No	321 (73)	111 (75.5)	107 (72.8)	103 (70.5)
Yes	118 (26.8)	35 (23.8)	40 (27.2)	43 (29.5)
Any history of drug treatment in lifetime				
No	309 (70.2)	104 (70.7)	111 (75.5)	94 (64.4)
Yes	130 (29.5)	42 (28.6)	36 (24.5)	52 (35.6)
Depression				
Not clinically depressed, ie, PHQ-9 score, ≤5	329 (74.8)	111 (75.5)	105 (71.4)	113 (77.4)
Mild depression, ie, PHQ-9 score, 5-9	86 (19.5)	30 (20.4)	32 (21.8)	24 (16.4)
At least moderate depression, ie, PHQ-9 score, ≥10	25 (5.7)	6 (4.1)	10 (6.8)	9 (6.2)
Anxiety				
No anxiety disorder, ie, GAD-7 score, <8	425 (96.6)	145 (98.6)	141 (95.9)	139 (95.2)
Probable anxiety disorder, ie, GAD-7 score, ≥8	15 (3.4)	2 (1.4)	6 (4.1)	7 (4.8)
Current ART use				
No	2 (0.5)	1 (0.7)	1 (0.7)	0
Yes	438 (99.5)	146 (99.3)	146 (99.3)	146 (100)
Time on ART, median (IQR), y	5.1 (2.8-8.0)	5.0 (2.5-7.8)	6.2 (3.1-8.1)	5.0 (3.0-7.7)
Missed at least one ART tablet in past 1 mo, d^c^				
0	352 (80.0)	122 (83.0)	119 (81.0)	111 (76.0)
1	49 (11.1)	15 (10.2)	13 (8.8)	21 (14.4)
2	23 (5.2)	4 (2.7)	9 (6.1)	1 (0.7)
3-7	13 (3.0)	4 (2.7)	5 (3.4)	4 (2.7)

Abbreviations: ART, antiretroviral therapy; AUDIT, Alcohol Use Disorders Identification Test; GAD-7, Generalized Anxiety Disorder–7 score; IQR, interquartile range; PHQ-9, Patient Health Questionnaire–9 score.

^a^ Defined by the Mini-International Neuropsychiatric Interview version 5.0.0.

^b^ Data were missing for 1 participant due to not knowing.

^c^ Data were missing for 3 participants (1 participant, due to not knowing; 2 participants, not currently receiving ART).

**Figure.**
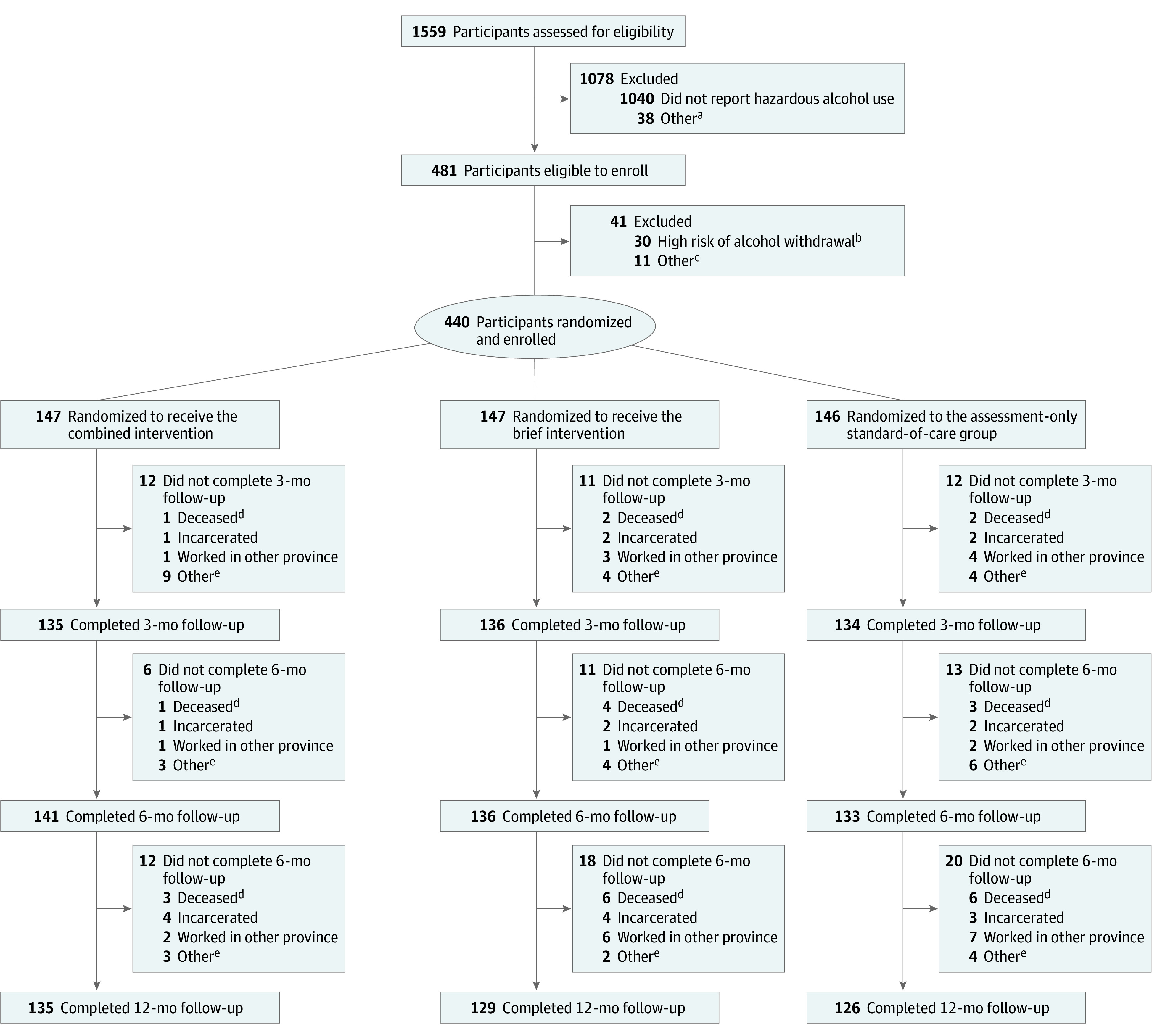
Trial Flowchart ^a^Other reasons include currently participating in another HIV, drug use, or alcohol program; planning to move from province in next 24 months; and unwilling to adhere to program. ^b^High risk of alcohol withdrawal was defined as Clinical Institute Withdrawal Assessment of Alcohol Scale score of at least 10. Participants could rescreen if they returned with a Clinical Institute Withdrawal Assessment score less than 10. ^c^Other reasons included not completing baseline assessment; not completing baseline laboratory testing; and other. ^d^Number of deaths is cumulative. ^e^Other reasons included could not be contacted; refused to return; and other.

### Baseline Characteristics

Demographic and clinical characteristics at baseline were comparable across groups (Table 1). Overall, 184 (41.2%) had current alcohol dependence or alcohol abuse. Most had a history of injection drug use (356 [80.9%]), although only approximately one-quarter (118 [26.8%]) had injected drugs in the past 3 months. Many participants (111 [25.2%]) had clinical depression.

### Intervention Uptake

In the combined intervention group, 144 participants (98.0%) attended the first individual session, 133 (90.5%) attended at least 3 sessions, and 112 (76.2%) attended all 6 individual sessions. In addition, 123 (83.7%) attended at least 1 optional group session, and 112 (76.2%) attended all 3 optional group sessions. Individual sessions lasted a mean of 51 minutes (range, 29-76 minutes), and group sessions lasted a mean of 65 minutes (range, 60-70 minutes).

Among participants in the brief intervention group, 140 (95.2%) attended at least 1 in-person session, and 124 (84.4%) attended all 4 sessions. The initial session was a mean of 57 minutes in duration (range, 27-78 minutes), and the second was a mean of 31 minutes (range, 24-38 minutes). Both phone sessions lasted a mean of 9 minutes (range, 6-12 minutes). In addition to high rates of session attendance, counselors had high fidelity to the combined intervention manual (mean [SD] YACS score,^28^ 5.05 [0.54]) and the brief intervention manual (mean [SD] YACS score, 5.45 [0.31]).

### Percentage of Days Abstinent

At baseline, the mean (SD) percentage of days abstinent in the past 30 days was 40% (1.6%), with no significant differences across arms (Table 2). At the 3-month visit, the proportion increased to a mean (SE) of 61% (3.1%) in the combined intervention group, 67% (2.9%) in the brief intervention group, and 44% (3.2%) in the SOC group (Table 2) (Cohen *d* for difference for combined intervention vs SOC: 46%; 95% CI, 22% to 70%; for brief intervention vs SOC: 64%; 95% CI, 40% to 88%; for combined intervention vs brief intervention: –18%; 95% CI, –41% to 6%) (eTable 3 in Supplement 2). This difference was sustained through the 12-month visit, at which the mean (SE) percentage of days abstinent was 65% (3.1%) in the combined intervention group and 65% (3.2%) in the brief intervention group compared with 50% (3.4%) in the SOC group (Table 2) (Cohen *d* for difference for combined intervention vs SOC and brief intervention vs SOC: 39%; 95% CI, 15% to 64%; for combined intervention vs brief intervention: 0%; 95% CI, –24% to 24%) (eTable 3 in Supplement 2).

**Table 2.** Percentage of Days Abstinent From Alcohol in the Last 30 Days, by Trial Group and Visit

Outcome	Overall	Combined intervention	Brief intervention	Standard of care	*P* value
Baseline					
Attended visit, No.	440	147	147	146	NA
Days abstinent, mean (SE), %	40 (1.6)	37 (2.8)	41 (2.8)	41 (2.7)	.60
3-mo follow-up					
Attended visit, No.	404	135	135	134	>.99
Days abstinent, mean (SE), %	58 (1.8)	61 (3.1)	67 (2.9)	44 (3.2)	<.001
6-mo follow-up					
Attended visit, No.	409	141	135	133	.22
Days abstinent, mean (SE), %	59 (1.9)	62 (3.1)	65 (3.2)	49 (3.3)	<.001
12-mo follow-up					
Attended visit, No.	390	135	129	126	.29
Days abstinent, mean (SE), %	60 (1.9)	65 (3.1)	65 (3.2)	50 (3.4)	.002
Among those who attended the follow-up visit, baseline percentage of days abstinent from alcohol use in the last 30 d, mean, %					
3-mo follow-up	40 (1.7)	39 (2.9)	42 (2.9)	40 (2.8)	.78
6-mo follow-up	40 (1.6)	37 (2.8)	41 (2.8)	41 (2.8)	.55
12-mo follow-up	40 (1.7)	38 (2.9)	40 (2.9)	42 (2.9)	.67

Abbreviations: NA, not applicable.

### Viral Load

The percentage of participants achieving viral suppression (ie, <20 copies/mL) was similar at 3 and 6 months, but at 12 months, mean (SE) viral suppression was more common in the combined intervention group (83% [3%]) and the brief intervention group (92% [2%]) than in the SOC group (77% [4%]) (Table 3). Given that the mean (SE) percentage of virally suppressed participants at baseline was 88.4% (2.6%) in the brief intervention group vs 80.1% (3.3%) in the SOC group, we standardized all arms to the mean baseline level of 84% virally suppressed (Table 4). After standardizing covariates, at 12 months the brief intervention group still had the highest mean (SE) percentage of participants with viral suppression (89.2% [3.1%]) followed by the combined intervention group (83.1% [3.1%]) and SOC (78.1% [3.7%]) (Table 4) (brief intervention vs SOC: difference, 11%; 95% CI, 2% to 20%; combined intervention vs SOC: difference, 5% 95%, CI, –5% to 15%; combined intervention vs brief intervention: difference, –6%; 95% CI –13% to 1%) (eTable 4 in Supplement 2).

**Table 3.** Percentage of Participants With Viral Suppression by Trial Group and Visit^a^

Outcome	Overall	Combined intervention	Brief intervention	Standard of care	*P* value
Baseline					
Attended visit, No.	440	147	147	146	NA
Virally suppressed, % (SE)	84.1 (1.7)	83.6 (3.0)	88.4 (2.6)	80.1 (3.3)	.13
3-mo follow-up					
Attended visit, No.	403	135	136	132	.79
Virally suppressed, % (SE)	85.6 (1.7)	81.4 (3.3)	89.7 (2.6)	85.6 (3.1)	.15
6-mo follow-up					
Attended visit, No.	410	141	136	133	.21
Virally suppressed, % (SE)	85.9 (2.9)	86.5 (2.9)	87.5 (2.8)	83.4 (3.2)	.63
12-mo follow-up					
Attended visit, No.	390	135	129	126	.29
Virally suppressed, % (SE)	84	83 (3)	92 (2)	77 (4)	.003
Among those who attended each follow-up visit, baseline proportion with viral suppression, %					
3-mo follow-up	84.3 (1.8)	82.2 (3.3)	91.2 (2.4)	79.6 (3.5)	.01
6-mo follow-up	84.6 (1.8)	83.7 (3.1)	90.4 (2.5)	79.7 (3.5)	.03
12-mo follow-up	84.9 (1.8)	83.7 (3.2)	91.5 (2.5)	79.4 (3.6)	.01

Abbreviations: NA, not applicable.

^a^ Viral suppression defined as less than 20 copies of HIV-1 RNA per milliliter.

**Table 4.** Percentage of Participants With Viral Suppression With Covariate Standardized to Have the Same Baseline Percentage of Viral Suppression^a^

Outcome	Combined intervention	Brief intervention	Standard of care	*P* value
Baseline^b^				
Attended visit, No	147	147	146	NA
Virally suppressed, %	84	84	84	>.99
3-mo follow-up				
No. attended visit	135	136	132	.79
Proportion virally suppressed, % (SE)	82.2 (3.0)	86.6 (3.0)	87.1 (2.7)	.43
6-mo follow-up				
Attended visit, No.	141	136	133	.21
Proportion virally suppressed, % (SE)	86.7 (2.6)	84.6 (3.1)	84.8 (3.0)	.84
12-mo follow-up				
Attended visit, No.	135	129	126	.29
Proportion virally suppressed, % (SE)	83.1 (3.1)	89.2 (3.1)	78.1 (3.7)	.06

Abbreviations: SE, standard error.

^a^ Viral suppression defined as less than 20 copies of HIV-1 RNA per milliliter.

^b^ Each arm’s covariate has been standardized to have 84% of participants virally suppressed at baseline.

The observed effect sizes for abstinent days were within the range of those planned in the protocol to have enough power. For viral load, the observed effect for BI vs SOC was similar to the planned power, although the observed combined intervention vs SOC effect was smaller than what was planned (eTable 5 in Supplement 2).

### Secondary Outcomes

Relative to SOC, both the brief and combined interventions reduced the mean (SE) number of drinks per drinking day (4.2 [0.3] vs 3.4 [0.3] and 2.9 [0.2], respectively) at 12 months (eTable 6 and eTable 7 in Supplement 2). Likewise, compared with SOC, the mean (SE) number of heavy drinking days was lower among participants in the brief and combined interventions (6.7 [1.0] vs 3.7 [0.7] and 3.4 [0.7], respectively) at 12 months (eTable 8 and eTable 9 in Supplement 2).

### Phosphatidylethanol Validation of Self-Reported Abstinence

As anticipated, having a phosphatidylethanol level of less than 8 ng/mL and self-reporting complete abstinence in the past 3 weeks were strongly associated. At baseline, the odds ratio was 9.8 (95% CI, 2.3 to 40.9); at 3 months, 5.8 (95% CI, 2.9 to 11.6); and at 12 months, 6.4 (95% CI, 3.2 to 12.7) (eTable 10 in Supplement 2).

## Discussion

In this randomized clinical trial, we observed that alcohol reduction can be achieved through a brief intervention, leading to substantial improvement in viral suppression. Both integrated alcohol reduction interventions, the combined intervention and brief intervention, led to increased percentage of days abstinent from alcohol compared with standard of care; this effect was sustained during 12 months. Notably, self-reported percentage of days abstinent using the timeline follow-back interview was validated using the alcohol biomarker phosphatidylethanol. In the 12-month visit, the proportion of virally suppressed participants increased by 5% in the brief intervention group and dropped by 6% in the SOC group. In the combined intervention arm, the proportion of patients who had viral suppression stayed approximately the same across visits. The 11% difference in viral suppression between the brief intervention and SOC groups more than halved the proportion of participants who were not virally suppressed and has major clinical implications for transmission in this high-risk population.

As described elsewhere,^29^ the combined intervention is more resource-intensive than the brief intervention ($30 additional cost per participant); however, the brief intervention was at least as, if not more, effective than the combined intervention on biologically validated alcohol measures and viral suppression. Part of the explanation for our findings may be that, given the lack of alcohol reduction programs in Vietnam, even 2 in-person sessions and 2 telephone sessions of alcohol reduction counseling can increase awareness of the harms of alcohol and teach coping skills to manage high risk moods and/or situations for alcohol use. Importantly, these results underscore that a brief intervention provided by paraprofessional counselors effectively reduced alcohol consumption and increased viral suppression among people with hazardous or heavy drinking being treated in ART clinics. Practical implications include integrating the brief intervention into ART clinics and screening for ART clients with hazardous drinking. Our findings highlight the need for a rigorously evaluated implementation study in multiple settings to evaluate whether the same results could be achieved in real-world settings in a wide variety of low- to middle-income countries.

Heavy alcohol use among PWH is prevalent and is associated with lower ART adherence, lower rates of viral suppression, and increased sexual and injecting risk behaviors.^10,11,16^ People who drink alcohol excessively may be more likely to become HIV infected.^21^ But higher rates of drinking among PWH is likely due in part to coping with the stress of HIV-related stigma and negative life events, such as an HIV diagnosis, as well as lack of social support.^21,34^ Training in alternative means of coping may both reduce alcohol use and enhance well-being. But alcohol treatment has rarely been incorporated into HIV care settings. In the United States, a stepped alcohol treatment intervention for patients with HIV and alcohol use disorder incorporating a physician-managed medication-based intervention, motivational enhancement therapy, and referral to intensive outpatient or residential treatment led to improvements in the proportion of days abstinent and the proportion of patients with an undetectable HIV viral load at 52 weeks.^35^ We showed the effects of 2 counseling-only interventions on these same outcomes at 52 weeks, which, unlike physician-managed medication-based interventions, can be realistically scaled in low-resource settings. Specifically, both the combined and brief interventions have a highly structured format that are manualized and can be delivered by trained paraprofessionals. Notably, alcohol reduction and associated improvement in viral load were achieved in patients who were drinking at hazardous or heavy levels but were not seeking alcohol treatment.

At 12 months, viral suppression was significantly higher in the brief intervention group compared with the SOC group; however, the percentage of virally suppressed participants was also higher in the brief intervention group than the SOC group at baseline. After standardizing both baseline levels of viral suppression and dropout rates over time, the brief intervention group still had a significantly higher level of viral suppression compared with the SOC group. This suggests that the brief intervention reduced alcohol use, which in turn improved ART adherence, leading to greater viral suppression compared with SOC.

Our study included only individuals receiving ART and did not exclude those who had already achieved viral suppression; therefore, a high proportion of our study population already had viral suppression at baseline. We anticipate that including people who are not receiving ART and excluding those who had achieved viral suppression would increase the effect of the interventions on viral suppression. In settings where viral suppression is already high, it is important to reach the final non–virally suppressed 10% to 15% of patients, who are often the most challenging to engage in HIV care.

Self-reported alcohol abstinence was a primary outcome, but other measures of alcohol reduction may also achieve viral suppression. Comparing the proportion of patients who reduced alcohol consumption between the SOC and combined intervention groups and the SOC and brief intervention groups at 12 months is important to understand how the interventions may operate to affect viral suppression. We found that both number of drinks per drinking day and number of heavy drinking days at 12 months were reduced in the combined and brief intervention groups compared with the SOC group, providing additional support for the effectiveness of the combined and brief interventions.

### Limitations and Strengths

This study has limitations. Self-reported alcohol abstinence is susceptible to social desirability bias. To assess the extent of this bias, we compared phosphatidylethanol level of less than 8 ng/mL, the established cut-off for abstinence, to self-reported alcohol abstinence. Only 1% to 6% reported no alcohol use in the past 21 days but had phosphatidylethanol levels of greater than 8 ng/mL, suggesting social desirability at play. However, we saw a similar percentage of those with phosphatidylethanol levels of less than 8 ng/mL who reported alcohol use in the past 21 days, suggesting possible imperfections in either or both measures that work in both directions. Therefore, while there was a strong association between phosphatidylethanol levels of less than 8 ng/mL and self-reported alcohol abstinence, it is still unclear how to best use phosphatidylethanol.

Most participants in this study were men. This was expected, given that 65% of PWH are men^36^ and most individuals with alcohol use disorder in Vietnam are men.^36^ Therefore, we anticipate that our findings will generalize to our target population in Vietnam, although they may not be generalizable to other contexts in which women account for a greater proportion of PWH and people with alcohol use disorders

This study also has several strengths. The interventions were designed to be scalable. Both interventions were conducted in the very clinic settings in which they could be implemented during scale-up. By integrating into an existing structure and enrolling current ART clients, we had the opportunity to evaluate the interventions in real-world clinic settings to directly inform future scale-up.^37^ The role of counselors can likely be fulfilled by peers, social workers, counselors, or clinicians. The counselor role did not require a high level of education, and some counselors did not have a bachelor’s degree. Indeed, lay counselors have effectively delivered psychological treatment for men with alcohol dependence in primary care settings in India.^38^ Uptake of both interventions was excellent. Attendance was high compared with other trials of the combined and brief interventions^38^ and was relatively higher for the brief intervention, which was expected given the lower burden on the participants (ie, 6 in-person sessions vs 2 in-person sessions plus 2 brief telephone sessions). Duration of sessions, including the brief intervention telephone sessions, was also longer than expected, reflecting high engagement during the sessions. Counselor fidelity to the intervention manual was also acceptable.

## Conclusions

In summary, the brief intervention, which combined in-person and telephone sessions, and the combined intervention, which included individual and group sessions, increased the percentage of days abstinent from alcohol (confirmed using phosphatidylethanol), decreased the number drinks per drinking day, and decreased the number of heavy drinking days. These effects were sustained through 52 weeks. Furthermore, the brief intervention increased viral suppression at 52 weeks. Based on the strength of the brief intervention compared with the combined intervention as well as the lower resources required to sustain the brief intervention compared with the combined intervention, implementation of the brief intervention should be a priority in other populations of ART clients.

## Notes

Supplement 1. **Trial Protocol and Statistical Analysis Plan**

Supplement 2. **eTable 1. **Number of Participants Enrolled in the Study From Each ART Clinic, Thai Nguyen Province, Vietnam **eTable 2. **Descriptions of Each Alcohol Reduction Intervention Evaluated **eTable 3. **Cohen *d* for Difference Among Groups for Mean Percentage of Days Abstinent From Alcohol in the Last 30 Days **eTable 4. **Difference Among Groups for Percentage Viral Suppression in the Last 30 Days **eTable 5. **Observed Effect Size vs Planned Power for Primary Outcomes **eTable 6. **Mean Number of Drinks per Drinking Day in the Last 30 Days, by Groups and Visits **eTable 7. **Cohen *d* for Difference Among Groups for Mean Number of Drinks per Drinking Day in the last 30 Days **eTable 8. **Mean Number of Heavy Drinking Days in the Last 30 Days, by Groups and Visits **eTable 9. **Cohen *d* for Difference Among Groups for Mean Number of Heavy Drinking Days in the last 30 Days **eTable 10. **Self-reported Alcohol Abstinence in the Last 3 weeks and Phosphatidylethanol Levels Less Than 8 ng/mL

Supplement 3. **Data Sharing Statement**
