# Errors in Table 3 and Table 4

**DOI:** 10.1001/jamanetworkopen.2020.26150

**Published:** 2020-10-14

In the Original Investigation titled “Effect of 2 Integrated Interventions on Alcohol Abstinence and Viral Suppression Among Vietnamese Adults With Hazardous Alcohol Use and HIV: A Randomized Clinical Trial,”^1^ published September 18, 2020, there were errors in Table 3 and Table 4. In Table 3, the number of patients who attended the 3-month follow-up, the proportion of patients virally suppressed at 3 months, and the baseline proportion of patients with viral suppression were incorrect; in Table 4, the number of patients who attended the 3-month follow-up and the proportion of patients virally suppressed at 3 months were incorrect. In Table 3, a total of 405 patients, 137 of whom were in the combined intervention group, attended the 3-month follow-up (*P* = .66). The proportion (SE) of patients with viral suppression was 85.7% (1.7%) overall and 81.8% (3.3%) in the combined intervention group (*P* = .15). The baseline proportion of patients with viral suppression at 3 months was 84.4% (1.8%) overall and 82.5% (3.2%) in the combined intervention group. In Table 4, a total of 137 participants in the combined intervention group attended the 3-month follow-up; the proportion (SE) of patients with viral suppression was 82.5% (3.2%) (*P* = .45). In both tables, 2 participants who did not complete their 3-month visit had 3-month viral load data. This article has been corrected.^1^
