# Supplementary material for: Effect of 2 Integrated Interventions on Alcohol Abstinence and Viral Suppression Among Vietnamese Adults With Hazardous Alcohol Use and HIV: A Randomized Clinical Trial
Source: JAMA Netw Open. 2020 Sep 18;3(9):e2017115. doi: 10.1001/jamanetworkopen.2020.17115 (PMC7501538; doi:10.1001/jamanetworkopen.2020.17115)
Supplement: Supplement 1. — Trial Protocol and Statistical Analysis Plan [file jamanetwopen-e2017115-s001.pdf]

REDUCING HAZARDOUS ALCOHOL USE & HIV VIRAL LOAD: AN RCT IN ART CLINICS IN VIETNAM (REDART)

Protocol and Statistical Analysis Plan

Reducing hazardous alcohol use & HIV viral load: an RCT in ART clinics in Vietnam (REDART)

TABLE OF CONTENTS

FINAL PROTOCOL (Version 2.0) ………………………………………………………………… 3

SUMMARY OF PROTOCOL CHANGES ………………………………………………………….. 36

STATISTCAL ANALYSIS PLAN ………………………………………………………………....... 37

PROTOCOL

Study Title: Reducing hazardous alcohol use & HIV viral load: an RCT in ART clinics in Vietnam (REDART)
PI: Vivian F. Go, PhD
Sponsor: NIDA
UNC IRB No.: 14-0225
Version Number / Date: Version 2.0 / 30 November 2016

SCHEMA

Purpose

To conduct a three-arm randomized controlled trial (RCT) among hazardous and heavy drinking HIV-infected ART clinic patients in Thai Nguyen, Vietnam, comparing the effects of two evidence-based interventions [a brief intervention vs. a combined intervention which draws from (motivational enhancement therapy (MET) and cognitive behavioral therapy (CBT)] both against each other and compared with an assessment-only control, in order to understand the relative effectiveness of each intervention in improving both alcohol- and HIV-related outcomes and provide insight into the optimal application of alcohol programs in resource-limited settings.

Study population

HIV-infected ART clinic patients with hazardous or heavy alcohol use in Thai Nguyen Province ART clinics.

Design

This is a three-arm randomized controlled trial (RCT) comparing the effects of two evidence-based interventions [a brief intervention (BI) vs. a combined intervention which draws from motivational enhancement therapy (MET) and cognitive behavioral therapy (CBT)] both against each other and compared with an assessment-only (standard of care) control. Individual participants will be randomized to the study arms in a 1:1:1 ratio. Longitudinal qualitative interviews will also be conducted among a subset of participants and all counselors from both intervention arms.

Study Size and Procedures

There are three main phases of this study: 1) The Formative Phase; 2) the Pilot Phase; and 3) the RCT Phase.

In the **Formative Phase**, in-depth interviews will be conducted among 40 participants. The results from these in-depth interviews will inform the cultural adaptation of the interventions, and provide important context in which alcohol use occurs in Vietnam.

The **Pilot Phase** will be conducted among up to 80 participants. Specifically, we will pilot intervention sessions among up to 40 participants (20 per intervention), and the baseline survey among up to 40 participants (up to 20 for cognitive interviews on select sections, and up to 20 for the full survey). After each piloting session, we will ask the participant open-ended questions regarding what they thought about each intervention session or survey they received. The purpose of the pilot phase is to inform investigators on which parts of the intervention worked and what parts need to be refined, and which parts of the survey need to be modified or omitted to fit the Vietnamese context.

In the **RCT Phase**, 441 HIV clinic patients with hazardous drinking in Thai Nguyen Province ART clinics will be randomized to receive the brief intervention (2 face-to-face sessions and 2 booster phone sessions total), the combined intervention (6 face-to-face sessions and 3 optional group sessions), or an assessment-only control condition. Each participant will have 3, 6, and 12-month follow-up assessments.

Treatment Regimen Individual study participants will be randomized to one of three study arms at a ratio of 1:1:1 (brief intervention: combined intervention: assessment-only). All participants will receive standard of care. Participants in the **brief intervention** will receive 2 intervention sessions each, plus 2 brief booster phone sessions. Participants in the **combined intervention** will receive 6 sessions total and up to 3 optional group sessions.

Study Duration Study activities will span across 5 years. The first 12 months will be dedicated to obtaining approvals, training staff, conducting formative research, adapting and piloting the interventions, developing the protocol, questionnaires, manuals, and SOPs, and translating study materials. Recruitment, screening and enrollment will be completed over approximately 12 months. Participants will be followed-up for 12 months (3, 6, and 12 months follow-up visits). All participants will end study participation after completion of their 12-month follow-up assessment or at the end of their 12-month visit allowable window period.

Primary Objectives

Aim 1: To conduct a three-arm randomized controlled trial (RCT) among hazardous and heavy drinking, HIV-infected clinic patients in Vietnam comparing the effects of two evidence-based, manually guided, individually delivered interventions: 1) a Brief Intervention (BI) and 2) Combined MET+CBT Intervention, both against each other and compared with an assessment-only control.

Aim 2: To measure the incremental cost-effectiveness of BI and the combined intervention as compared to current counseling services offered in ART clinics in Vietnam.

1.0 BACKGROUND AND RATIONALE

The prevalence of heavy alcohol consumption by people who inject drugs (PWID) ranges from 11% to 57%[1-4] and PWID have very high rates of Hepatitis B (HBV) and Hepatitis C (HCV) infection.[5] Alcohol hastens the progression of liver disease related to HBV and HCV,[6] is associated with liver-related mortality among HIV/HCV co-infected persons[7] and may accelerate HCV progression.[8] Hazardous alcohol use among HIV-infected PWID is independently associated with decreased ART adherence and viral suppression[9] as well as sharing needles/syringes, having multiple sex partners and engaging in unprotected sex, behaviors which are known risk factors for HCV and HIV acquisition and transmission.[10-13]

Effective strategies to reduce hazardous and/or heavy drinking among HIV-infected individuals are critical given the serious adverse health consequences of alcohol in this population, especially decreased adherence to antiretroviral therapy[10,14-17] and decreased virologic suppression.[9] Alcohol use is also associated with elevated sexual and injection risk behaviors that increase the likelihood of HIV transmission.[18-20] Despite the known adverse health consequences of alcohol use, hazardous and heavy alcohol consumption are frequently not assessed or addressed in HIV health-care settings. Asia has the second largest HIV epidemic globally with an estimated 4.9 million individuals infected with HIV.[21] Prevalent hazardous drinking among PWID and other risk groups in Asia has challenged HIV prevention efforts as well as the clinical management of HIV/AIDS care and treatment services.[14,15]

In Vietnam, where the HIV epidemic is concentrated among PWID, who are often co-infected with HCV/HBV, we found that hazardous alcohol use is widespread, reflecting cultural norms around drinking and accessibility of alcohol. Among HIV-infected PWID, previous studies found a high prevalence of hazardous drinking (20%) and a high rate of HBV/HCV co-infection (99%). Hazardous drinking is significantly associated with increased progression of liver disease among HIV-infected individuals who are co-infected with HBV and HCV,[22] further underscoring the need to address hazardous alcohol use among HIV clinic patients in this setting. Two psychosocial interventions, Motivational Enhancement Therapy (MET) and Cognitive Behavioral Therapy (CBT), have been shown to be highly effective in reducing alcohol use among people with hazardous/heavy alcohol use in US settings.[23] Both of these theoretically distinct interventions have potential applicability to the Vietnamese context. While CBT is skill-based and more comprehensive, MET seeks to impact behavior through internally motivated change and is brief and potentially less resource intensive. Understanding the relative effectiveness of each intervention in improving both alcohol- and HIV-related outcomes will provide insight into the optimal application of alcohol programs in resource-limited settings.

In order to develop and deliver the interventions in a culturally appropriate way, it is important that we understand the cultural context of alcohol use in Vietnam. We propose to conduct formative research to better understand the norms and context surrounding alcohol use in Vietnam, in general and among HIV-infected persons.

2.0 STUDY OBJECTIVES, AIMS AND HYPOTHESES

Our primary objective is to conduct a three-arm randomized controlled trial (RCT) among hazardous and heavy drinking HIV-infected ART clinic patients in Thai Nguyen, Vietnam, comparing the effects of two evidence-based interventions [a brief intervention vs. a combined intervention, both of which draw from (motivational enhancement therapy (MET) and cognitive behavioral therapy (CBT)] both against each other and compared with an assessment-only control, in order to understand the relative effectiveness of each intervention in improving both alcohol- and HIV-related outcomes and provide insight into the optimal application of alcohol programs in resource-limited settings.

Specifically, our study has two aims with corresponding hypotheses:

Aim 1: To conduct a three-arm randomized controlled trial (RCT) among hazardous and heavy drinking, HIV-infected clinic patients in Vietnam comparing the effects of two evidence-based, manually guided, individually delivered interventions (brief intervention (BI) and combined intervention) both against each other and compared with an assessment-only control. This aim will assess the following hypotheses:

1a. BI and the combined intervention will each be more effective than an assessment only arm on percent days alcohol abstinent and percent virally suppressed at the 12-month assessment [primary outcomes].
1b. BI will be equivalently effective to the combined intervention on percent days alcohol abstinent [primary outcome].
1c. The effect of BI and the combined on abstinence and viral suppression will be mediated by alcohol readiness to change and/or skill acquisition [secondary outcomes].
1d. The combined intervention will be more effective than BI on abstinence and viral suppression separately among participants with more severe alcohol use, injection drug users and participants with depressive symptoms [effect modifiers].

Aim 2: To measure the incremental cost-effectiveness of BI and the combined intervention as compared to current counseling services offered in ART clinics in Vietnam. Comprehensive costing will be performed in each clinic, health utility will be assessed at each visit and a Markov model will be constructed to estimate the cost-effectiveness and cost-utility of the BI and the combined intervention provided through HIV clinics relative to the existing care condition. This aim will assess the following hypotheses:

2a. Alcohol reduction interventions (BI and the combined intervention) will be highly cost-effective compared to assessment, using a willingness-to-pay threshold of Vietnam's gross domestic product (GDP) per disability-adjusted life year (DALY) averted [primary outcome].
2b. BI will be highly cost-effective relative to the combined intervention [primary outcome].

3.0 STUDY DESIGN

The study will be conducted in Thai Nguyen province, Vietnam, and occur in three phases:

I. Formative Phase
II. Pilot Phase
III. RCT Phase

I. Formative Phase

The Formative Phase of this study will use qualitative methods for conducting and analyzing in-depth interviews to help us better understand the context in which drinking alcohol occurs in Vietnam, and the considerations and modifications we may need to make to our interventions in order to ensure that they are appropriately adapted to the culture and context of alcohol consumption in Vietnam. The in-depth interviews will be among hazardous drinkers who are either a) ART clients in one of the ART clinics in Thai Nguyen or b) who are people who inject drugs (PWID) that are not recruited from the ART clinics (whom we will refer to as general PWID).

II. Pilot Phase

The purpose of the Pilot Phase is to pilot the two interventions separately and conduct component testing, in order to inform investigators on what parts of the interventions work and what parts need to be clarified and/or refined. Component testing will consist of in-depth interviews conducted with each participant after the intervention is piloted with that participant. Up to 40 participants (up to 20 per intervention) will participate in the Pilot Phase activities.

Additionally, we will conduct cognitive interviewing to help us refine the baseline quantitative questionnaire, then pilot the baseline questionnaire with up to 40 participants.

III. RCT Phase

This is a 3-arm randomized controlled trial to evaluate the comparative effectiveness and cost-effectiveness of two evidence-based interventions, a brief intervention (2 sessions) and a combined intervention (6 sessions), both of which draw from Motivational Enhancement Therapy (MET) and Cognitive Behavioral Therapy (CBT), to reduce alcohol use and determine the impact on viral load among hazardous and heavy drinking HIV-infected ART clinic patients

in Vietnam. We will assess whether these two interventions improve outcomes compared to an assessment-only control.

The purpose of including the assessment control is to be able to isolate the effects specific to each intervention technique, independent of the baseline assessment. Participants in the assessment control arm will continue to receive all counseling sessions that are currently offered within the ART clinics (standard of care).

4.0 STUDY SETTING AND POPULATION

Setting. This study will be conducted in Thai Nguyen, a semi-urban province in North Vietnam located 75 km north of Hanoi, with a population of 1 million. Thai Nguyen has 12 government-run outpatient ART clinics (9 community clinics, 1 hospital clinic, and 2 prison clinics) with a total of about 3150 adult clients on ART. These clinics are the sole source of ART medications in the province. We will recruit participants from and conduct the study in the 7 outpatient ART clinics in Thai Nguyen with the highest numbers of ART clients (6 community clinics and 1 hospital clinic).

Study population. Study participants will be clients of the 7 selected ART outpatient clinics in Thai Nguyen. 70% of patients at these centers are currently on ART; the remaining 30% are HIV-infected, but were not eligible for ART (CD4 count <= 350) under MOH 2013 guidelines. MOH has recently updated their ART eligibility criteria to CD4 <=500. Fifty-six percent of patients on ART in Thai Nguyen are PWID, and of these individuals, 95% are male; among non-PWID patients, 66% are female. Active PWID will be referred to MMT clinics, which are co-located with ART clinics in Thai Nguyen and readily accessible to our participants. We will include all clients on ART in this trial who meet our eligibility criteria.

4.1 Study Sample

Overall, the study will have up to 561 hazardous drinkers, comprised of:

- 40 participants in the **Formative Phase**,
- up to 80 participants in the **Pilot Phase**, and
- 441 participants in the **RCT Phase**.

*Hazardous drinkers (for the **RCT Phase**, we will use AUDIT-C score ≥ 4 for men; AUDIT-C score ≥ 3 for women):* Study interviewers will introduce the project to each potential participant. For those who are interested and 18 years of age or older, **baseline written informed consent** will be obtained and then interviewers will complete the screening survey, which will include the WHO Alcohol Use Disorders Identification Test (AUDIT) survey. Responses from the screening survey will be used to assess participant eligibility in the RCT. The AUDIT-C score will be calculated by research staff shortly after baseline questionnaire completion, and clients with an AUDIT-C score ≥ 4 for men and ≥ 3 for women will be considered to have hazardous alcohol use.

Formative Phase

Up to 40 in-depth interviews will be conducted among up to 40 study participants who are hazardous drinkers, using AUDIT cutoff of ≥8 (one interview per participant). The Formative Phase participants will be made up of the following subgroups:

10 ART clients who are non-PWID females
10 ART clients who are non-PWID males
10 ART clients who are PWID (male or female)
10 General PWID (i.e., not recruited from the ART clinics; male or female)

Pilot Phase

We will pilot the intervention sessions with up to 40 participants (up to 20 per intervention) and pilot the quantitative questionnaire with up to 40 participants, who are ART clinic clients with hazardous drinking (AUDIT score >= 8). For the Pilot Phase, we will sample from ART clinics in provinces neighboring Thai Nguyen.

RCT Phase

The RCT phase will be conducted among 441 ART clinic clients with hazardous drinking (AUDIT-C score ≥ 4 for men; AUDIT-C score ≥ 3 for women) from the 7 ART outpatient clinics in Thai Nguyen. An individual has to be a registered client of an ART clinic to participate in the RCT Phase. We do not anticipate any crossover of patients to other ART clinics as patients register at one clinic and are not permitted to attend other clinics.

Participants enrolled in the RCT phase will have baseline and 3, 6, and 12-month follow-up assessments.

4.2 Inclusion and Exclusion Criteria

Formative Phase

Inclusion criteria for ART clients include:

1. being 18-49 years of age;
2. having an AUDIT score ≥ 8 (hazardous alcohol use);
3. being a current ART client at one of the Thai Nguyen ART clinics;
4. being capable of providing voluntary informed consent.

Inclusion criteria for general PWID include:

1. being 18-49 years of age;
2. having an AUDIT score ≥ 8 (hazardous alcohol use);
3. having injected drugs in the past 3 months;
4. being capable of providing voluntary informed consent.

Exclusion criteria for all participants include:

1. being unable to participate in study activities due to psychological disturbance, cognitive impairment or threatening behavior;
2. currently participating in other HIV, drug use or alcohol studies.

Pilot Phase

Inclusion criteria for participants are:

1. being 18-49 years of age;
2. having an AUDIT score ≥ 8 (hazardous alcohol use);
3. being a current ART client at one of the 7 selected Thai Nguyen ART clinics;
4. being capable of providing voluntary informed consent.

Exclusion criteria for participants are:

1. being unable to participate in study activities due to psychological disturbance, cognitive impairment or threatening behavior.

RCT Phase

Study interviewers will introduce the project to each client and will administer **baseline written informed consent** to those who are interested and are 18 years of age or older. After obtaining baseline consent, the interviewer will conduct the screening survey with the client. This will include the AUDIT survey items, which will be used to help determine eligibility for enrolling in the RCT.

Inclusion criteria for participants into the RCT are:

1) be a current client on ART at the clinic;
2) have an AUDIT-C score ≥ 4 for men; AUDIT-C score ≥ 3 for women (hazardous alcohol use);
3) be 18 years of age or older;
4) plan on residing in Thai Nguyen for the next 24 months.

Exclusion criteria for participants are:

1) unable to participate in study activities due to psychological disturbance, cognitive impairment or threatening behavior (study staff will assess sobriety);
2) unwilling to provide locator information;
3) unwilling to provide informed consent;
4) currently participating in other HIV, drug use or alcohol program, study, or intervention

4.3 Recruitment Process

Formative Phase

Participants for in-depth interviews will be identified using purposive sampling. So as not to recruit from the pool of potential participants for the RCT Phase, participants for the Formative Phase will be recruited from a neighboring province to Thai Nguyen.

Pilot Phase

Participants for the Pilot Phase will be identified by the ART clinic providers, who will refer interested clients to our study for more information on participating. Interested clients who contact us will first be administered a brief anonymous screener survey (see Screener-Pilot) to determine their eligibility. If they are determined to be eligible for the pilot phase, then we will administer informed consent and schedule their first visit. So as not to recruit from the pool of potential participants for the RCT Phase, participants for the Pilot Phase will be recruited from a neighboring province to Thai Nguyen.

RCT Phase

The 7 ART community clinics in Thai Nguyen currently have a range between 183-773 patients on ART at each site for a total of 3150 ART clients. In cooperation with the Thai Nguyen Center for Preventive Medicine (CPM), we will conduct meetings at each clinic to introduce the project and present our plans.

Community Advisory Board. We will create a Community Advisory Board (CAB) that represents a range of relevant stakeholders in the community, including ART clients, their families, and community and health leaders within Thai Nguyen. Board meetings will be held every 6 months during the planning, implementation and evaluation process to provide an update, to answer any questions and to obtain advice on key issues. Inclusion of community members early in the study is critical to securing community support and generating creative solutions to implementation challenges.

Recruitment. Recruitment of study participants will be conducted in potentially all 7 ART clinics in Thai Nguyen. All recruitment will be exhausted in one clinic before moving on to recruitment in the next clinic. The order of clinics to be recruited from will be random. HIV providers will introduce the study to patients and inform them that, if they are interested in participating, the patient may speak with study or clinic staff in the clinic lobby to find out more information. *The provider will not be aware of which patients choose to participate and which do not.* Interested patients at each clinic will be given information about the project by study or clinic staff and those interested will be invited for **baseline written informed consent** and screening at the baseline visit to determine eligibility.

4.4 Participation Rates, Retention and Tracing Procedures

RCT Phase

To minimize possible bias associated with missing visits and loss-to-follow-up, once a participant is enrolled into the RCT, the study site will make every effort to retain him/her for 12 months of follow-up.

Subjects will be given an appointment for their next follow-up visit at each assessment visit and will be reminded during sessions of their next appointment. Detailed locator information including phone numbers and place of residence will be collected although we anticipate few

challenges following participants as they visit the clinic at least monthly to receive ART medications. Nonetheless, to maximize cohort retention, we will apply the following procedures:

1) Using a computerized follow-up program to track scheduled and missed study appointments;
2) Updating locator information at every study visit;
3) Contacting study participants by telephone within 24 hours of a missed appointment;
4) Attempting home visits if contact by telephone is not successful; if the participant has changed his/her address, information is solicited (while maintaining confidentiality of the participant) from locator contacts provided initially by the participant;
5) If the participant has been detained in a DTC or a non-criminal justice facility or hospitalized, we will conduct the follow-up visit after release; and
6) Participants who become incarcerated will be followed up after release.

A participant is considered lost to follow-up if telephone contacts and 5 home visits fail, if the participant has moved out of Thai Nguyen Province during the follow-up window of the study, or if the participant died during the study period. If a participant leaves ART care, follow-up interviews will be conducted in a private place in the participant’s home, with the participant’s permission.

4.5 Participant Withdrawal and Termination

Participants may voluntarily withdraw from the study for any reason at any time. The investigators may also withdraw participants from the study in order to protect the participant’s safety.

Participants may also be withdrawn from the study if the study sponsor, UNC or local IRBs or the DSMB terminate the study prior to study completion.

Participants will be considered terminated from the study if the participant actively withdraws or dies during the course of the study.

5.0 STUDY INTERVENTIONS

All participants in each of the 3 study arms will continue to receive the standard of care provided by their ART clinic.

The interventions will be implemented over a 3-month period. Sessions will be audiotaped with participant’s permission to ensure intervention fidelity.

5.1 Combined Intervention (also referred to as the MET+CBT Intervention)

The Combined (MET+CBT) Intervention comprises a total of 6 individual face-to-face sessions and 3 optional group sessions for each participant assigned to this arm, with individual sessions occurring approximately 1 week apart.

The MET+CBT Intervention combines aspects from both Motivation Enhancement Therapy (MET) and Cognitive Behavioral Therapy (CBT). It uses a client-centered, motivational interviewing approach and focuses on skills-building for alcohol use behavior change, including drinking refusal skills, skills to cope with and manage cravings and triggers, and developing positive thoughts and attitudes. It also includes review of drinking patterns and harmful effects of drinking.

The rationale for combining MET and CBT intervention programs into one overall intervention was based on our formative research results, which indicated that using MET and CBT separately, as they are used in the US, would not be culturally appropriate in the Vietnamese context. Therefore, investigators culturally adapted components of MET and CBT into this combined intervention to be more applicable to alcohol use behaviors in the Vietnamese context.

Additionally, there will be three optional group session(s) that participants assigned to the combined intervention arm may attend anytime after they are enrolled into the study. The group sessions are informal and may be scheduled according to the group of participants' availability, are not subject to the 12-week intervention window, and may take place anytime within the duration of the study.

There will be a 12-week window after participant's randomization into the study for the participant to complete their intervention sessions.

5.2 Brief Intervention

The Brief Intervention (BI) comprises a total of 2 individual face-to-face sessions and 2 individual booster phone sessions for each participant assigned to this arm. The face-to-face sessions will occur approximately 1 month apart, and a phone session will occur 2-3 weeks after each face-to-face session. The Brief Intervention (BI) consists of 2 individual sessions and 2 booster phone sessions delivered by a trained counselor and is based on Project Treat. Content of BI sessions includes review of drinking patterns, harmful effects of drinking, and alcohol use behavior change strategies. There will be a 12-week window after randomization into the study for the participant to complete their intervention sessions. The BI is based on Fleming's Project Treat, and it was culturally adapted to the Vietnamese context based on our formative research results.

5.3 Assessment-Only Control

147 participants will be enrolled and randomized to the assessment-only standard of care arm. This arm will include the baseline alcohol assessment, which can itself have an effect on behavior change.[79] By comparing the combined and brief interventions to assessment only, we

will be able to isolate the effects specific to each intervention technique, independent of the baseline assessment.

Table 5.1: Summary of assessment only and intervention arms

	**Assessment Only**	**Combined Intervention**	**Brief Intervention**
Standard of Care Services			
Harm reduction services	X	X	X
ART at any CD4 count	X	X	X
Referrals for substance use	X	X	X
Referrals for diagnosis and treatment of HBV, HCV, STIs, and TB	X	X	X
Face-to-face counseling sessions		6 sessions	2 sessions
Phone sessions			2 sessions
Group sessions (optional, up to 3)		X	

5.4 Counselors and Trainings

Counselors. Since our emphasis is on developing and disseminating programs that are both effective and widely applicable, it is important to have interventions that can be implemented by a range of counselors. Therefore, we will randomly assign counselors to each study arm, to ensure that our model and outcomes are not confounded by counselor preference or empathy skills. Counselors will be trained to competence in their intervention assignment. In order to assess the potential impact of counselor-specific characteristics, we will assess empathy among counselors[76] and will conduct post hoc analyses to assess if counselor characteristics are associated with participant outcomes.

Trainings. We will have a mobile team of counselors that will provide intervention sessions at each clinic. Thus, the same counselors will conduct the intervention sessions from clinic to clinic. In cooperation with the Thai Nguyen Center for Preventive Medicine (CPM), we will conduct meetings at each clinic to introduce the project and present our plans. Following the meetings, we will go through a 6-month period of intensive counselor trainings at a central location across all 7 sites, for each intervention separately (with simultaneous estimation of the costs of these intensive trainings). Initial training for both interventions will include four weeks of in-class sessions that will use modeling, role plays, video-taped feedback techniques. Each counselor will be videotaped in role-plays and rated for adherence and competency.

Potential counselors will be identified by the research team and invited to come in for initial basic trainings on HIV, addiction, alcohol use, and counseling skills, as appropriate. After the

basic trainings, the potential counselors will attend a series of trainings specific to only the intervention they would deliver. The core research staff and study investigators will conduct these trainings.

All costs of implementing the training (e.g., development of manuals, staff time, ongoing training requirements) will be collected prospectively by a third-party member of the study staff.

6.0 STUDY PROCEDURES

6.1 Study Procedures: Formative Phase

After being identified via purposive sampling, the potential participant will come in for 1 study visit. This one-time visit will include the screening, and if eligible, it will also include the in-depth interview.

During this visit, the potential participant will be administered oral consent, in which the participant will consent to the screening (referred to as the “short survey” in the oral consent forms) and if deemed eligible, the in-depth interview (referred to as the “longer interview” in the oral consent forms).

After the consent (and prior to the in-depth interview session), a pre-session questionnaire will be given to the participant to ask about basic socio-demographics and drug use and HIV history. Immediately following the pre-session questionnaire, the in-depth interview will be conducted. Trained interviewers will use in-depth interview guides developed by the investigators as the basis for the interviews.

The screening for each participant will take up to 30 minutes. Each in-depth interview will take approximately 60-90 minutes. All consents, screenings, and in-depth interviews will take place in private rooms at the project office in Thai Nguyen. This is not an HIV clinic. Trained research staff will conduct all consents, screenings and in-depth interviews.

Prior to the conducting of the in-depth interviews, we will conduct 9-13 stakeholder interviews among people who have key insights into community norms surrounding alcohol use in Vietnam. We will ask people who work in venues that serve alcohol and HIV providers about their broad (third-person) views about the cultural context of alcohol drinking in Vietnam in general and, for the HIV providers, the context of alcohol use among HIV patients in general. These will be one-time interviews; no identifiers will be collected, and we will not ask about personal details of the respondents nor ask about sensitive issues, but rather, will ask about broad views of the context of alcohol use in Vietnam. The information from these stakeholder interviews will be used to inform and refine the in-depth interview guides.

6.2 Study Procedures: Pilot Phase

In the Pilot Phase of this study, we will pilot the two interventions as well as the baseline quantitative survey. Up to 80 participants will participate in the Pilot Phase:

- Pilot and component testing of Combined Intervention (n=20)
- Pilot and component testing of Brief Intervention (n=20)
- Pilot and cognitive interviewing select baseline survey sections (n=20)
- Pilot of full baseline survey (n=20)

Piloting the interventions. We will conduct pilot testing of the two interventions among up to 40 participants (up to 20 participants per intervention) and use qualitative methods for conducting and analyzing in-depth interviews (see "Component Testing Guide" and "Component Testing Guide-Brief Intervention") that will assess how participants felt about the interventions. The results will inform how we may further adapt and refine the intervention to be most appropriate for this cultural context.

Piloting the survey. We will pilot the quantitative survey in two waves:

1) We will conduct cognitive interviews for 4 specific sections of the quantitative baseline survey, with up to 5 participants per section (total N=up to 20):

- Alcohol Abstinence Stigma
- Alcohol scales
- Patient Provider Trust
- Readiness to Change and Coping Skills

The scales from these 4 sections have either not been used in research studies in Vietnam previously or have been used only a few times in Vietnam (to the best of the investigators' knowledge). Therefore, cognitive interviewing of these sections will ensure that the questions are appropriate and make sense in the cultural context of northern Vietnam. We will use qualitative methods for conducting and analyzing in-depth interviews that will assess how participants felt about the survey questions.

2) Results from the cognitive interviews will be used to revise the survey. We will then pilot the full baseline survey and Computer-Assisted Personal Interview (CAPI) procedures with up to 20 ART clients with similar characteristics to our target population. The pilot will assess survey length, and check response distribution, item comprehension and data collection procedures. We will use qualitative methods for conducting and analyzing in-depth interviews that will assess how participants felt about the survey.

6.3 Assessment Visit Procedures: <u>RCT Phase</u>

Quantitative Assessments. Quantitative assessments will occur at baseline, 3, 6, and 12 months for all participants in the RCT Phase. All questionnaires will be administered through face-to-face interviews in a private room at the project facility with trained interviewers.

Participants who appear influenced by alcohol (assessed by the counselor and/or by Alco-sensor) or drugs or otherwise not lucid will be asked to return for an interview the next day. Study staff will encourage these participants not to drive a motorbike or car from the study site in that state.

6.3.1 Screening, Baseline Assessment and Enrollment (see also: Appendix 1)

At baseline, for each potential participant are (see also: **Appendix 2**):
- Independent **baseline written informed consent** for screening will be obtained before screening and baseline procedures are initiated.
- If the client is eligible and consents to participate in the RCT, the interviewer will administer independent **enrollment written informed consent** and **stored specimen informed consent** prior to enrollment and any randomization or follow-up procedures.

Baseline assessment will be performed at the ART clinics. Study interviewers will introduce the project to each client and, among those who are interested and are 18 years of age or older, interviewers will administer the **baseline written informed consent** to participate in the baseline questionnaire then administer the baseline questionnaire among those who consent.

The baseline assessment, a structured questionnaire, will consist of baseline risk assessment (e.g., AUDIT and other alcohol use items, drug use, sexual risk behaviors, injecting risk behaviors) and questions on topics including sociodemographics, self-reported adherence to ART, self reported HBV and HCV, depression, experiences with health care providers, experiences with violence, and process evaluation measures including perceptions and attitudes towards drinking alcohol. The baseline assessment will take approximately 1-2 hours to complete.

For Aim 2, the baseline and 3-month follow-up assessments will also include: (1) structured questions on costs borne to attend clinic (transportation, childcare, food, lost wages, etc.) based on prior evaluations of patient costs in Vietnam[82] and the World Health Organization's "Tool to Estimate Patients' Costs";[83] and (2) the health status measure EQ-5D-5L,[84] a five-item questionnaire (updated in 2011) designed to capture health utility that has been validated in Vietnamese individuals living with HIV/AIDS.[85] These assessments will allow us to collect one of the most comprehensive sets of data to date on HIV-infected patients' costs and quality of life in relation to HIV care and alcohol use in a disadvantaged, highly resource-constrained setting.

Trained interviewers who are not ART clinic staff will administer the questionnaires using computer assisted personal interviewing (CAPI) via tablets. The Timeline Followback (TLFB) alcohol data of the quantitative assessment, however, will be recorded on paper then entered manually into a database.

Procedures for decreasing risks from alcohol withdrawal:
In order to lower risks from alcohol withdrawal, ***before*** enrolling eligible participants into the trial, we will assess whether they are currently in alcohol withdrawal and should taper off before starting any study activities by implementing the following procedures:

1) We will conduct the CIWA with participants who are at high risk for withdrawal; specifically, we will conduct the CIWA with participants IF:
   1. The participant answered "Yes" to 3 or more of the survey questions D2a to D2g (the MINI questions); or
   2. The participant answered "Yes" to question D2b; or
   3. The participant had a history of alcohol withdrawal symptoms, especially if they have a history of requiring inpatient admission for detox, history of withdrawal seizures, and/or history of DT's (delirium tremens).
2) If participant's CIWA score ≥ 10, then the participant will receive the "Alcohol Taper Off steps" participant handout and the study physician will review these steps with the participant in-person. (Note: The rationale for providing these "Alcohol Taper Off steps" to participants is that alcohol withdrawal treatment with medications is extremely expensive and not a realistic option for most people in Vietnam.)
3) After the participant has tapered off, they may come back to the site to be administered the CIWA again. If their CIWA score is <10, then they may continue with procedures to enroll into the study and participate in the trial activities.

Enrollment. Among those who are eligible for enrollment into the RCT:

1) Enrollment eligibility will be confirmed using the **enrollment eligibility checklist** that must be reviewed and signed off by designated senior staff.
2) **Enrollment written informed consent** will be conducted. Specifically, interviewers will describe the RCT study objectives, procedures, risks and benefits to the eligible participants, and answer any questions. Interviewers will administer the **enrollment written informed consent** for the client to enroll in the RCT. Once written informed consent is obtained, the participant will be enrolled into the study.
3) Interviewers will collect locator information from the enrolled participant.
4) Trained laboratory staff will then collect baseline laboratory specimens from the enrolled participant.

Baseline and enrollment procedures:

1) **Baseline:** Among clients 18 years or older who are interested in participating
   - Baseline written informed consent
   - Baseline questionnaire
   - TLFB
   - Retrieving physical examination data from patient records
   - Enrollment eligibility checklist

2) **Baseline laboratory procedures:** Among clients eligible for enrollment only
   - Baseline stored specimen informed consent
   - Blood collection
   - Lab tests:
     - HIV viral load
     - CD4 count
     - HBsAg rapid test

- HCV rapid test
- PEth
- Plasma storage

3) **Return test results**
   - Referral for diagnosis and treatment of HBV, HCV

4) **Enrollment and Randomization**
   - Enrollment eligibility checklist – re-confirm eligibility
   - Enrollment informed consent
   - Enrollment stored specimen informed consent
   - Randomization (see details in next section, 6.3.2.)

Summary of baseline and enrollment procedures:

6.3.1.1 Screening:
1) Interviewer will conduct **Baseline informed consent** form to all participants
2) Collect participant's information for consented participants
3) Conduct screening survey for consented participants
4) Determine screening eligibility: based on eligibility of participants, conduct 6.3.1.2 *or* 6.3.1.3 for each participant; do *not* apply both 6.3.1.2 and 6.3.1.3 for the same participant.

6.3.1.2 Interview baseline survey for participants who are ineligible for the study after the screening survey:
1) Continue to interview baseline survey among ineligible participants
2) Complete baseline visit checklist and escort participants back to reception room.

6.3.1.3 Enrolment process for participants who are eligible for the study after the screening survey:
1) Before any further baseline procedures (including blood draw, TLFB, and baseline survey), interviewer will conduct 2 additional informed consent forms for eligible participants:
   - **Enrollment informed consent form**
   - **Stored specimen informed consent form**
2) Blood collection for consented eligible participants
3) Interview TLFB
4) Interview baseline survey

6.3.2 Randomization

We will complete the baseline visit procedures with ART clients and confirm enrollment eligibility prior to random assignment of individuals to study arms. Eligibility will be confirmed prior to randomization by using an enrollment eligibility checklist that must be reviewed and signed off

by designated senior staff. Once enrollment eligibility is confirmed, the individuals will be randomly assigned to one of the three study arms at a 1:1:1 ratio.

Eligible participants will be randomized at each clinic, one clinic at a time. A randomization schedule will be generated prior to the first enrollment, using permuted-block randomization with block size of 3, with study arms randomized at a 1:1:1 ratio. We will enroll and randomize participants at a clinic until we have screened all clients who are interested in participating in the study at that particular clinic. All eligible participants at one clinic will be randomized at that clinic before study staff move on to assess eligibility of participants at the next clinic. Trained core research staff will generate the randomization schedule using SAS. Specifically trained and designated staff will apply the randomization schedule to each enrolled participant.

The allowable window period between the screening day and randomization day is 30 days.

6.3.3 Follow-up Assessments

For those eligible to enroll in the RCT, we will administer **informed consent for enrollment and follow-up**, as well as a separate **enrollment stored specimen informed consent** (these consents will be administered one-time, within 30 days after the baseline visit when the participant was determined eligible). A total of 441 patients across participating ART clinics will be enrolled over a period of 12 months. Enrolled participants will be followed at 3, 6 and 12 months after baseline.

Follow-up assessment procedures:

- Confirm enrollment informed consent (prior to 3-month follow-up assessment)
- Confirm enrollment stored specimen informed consent (prior to 12-month specimen collection)
- Follow-up assessment
- TLFB
- Locator Information
- Blood collection
- Retrieving physical examination data from medical records
- Laboratory procedures
    - HIV viral load
    - Plasma storage
    - PEth collection
    - CD4 (at 12 months only)
- Cost-effectiveness assessment (at 3 months only)

All participants will be asked to complete a survey **at 3, 6, and 12 months** after baseline in a private room in the ART clinics. These visits will include a follow-up survey (including the health utility measure EQ-5D-5L), locator information update, blood collection to assess HIV-1 RNA level, and retrieval of physical examination data from medical records. Provision of viral load results will be provided on site in all three arms.

Allowable visit windows and missed visits: The visit window for a follow-up visit date is defined as the target day on which a visit should ideally occur. Target dates are based on the date of enrollment, which is the date of randomization (Day 0), and does not change even if a visit takes place before or after the target date. The allowable visit window is an extension of the target window and is the timeframe within which each visit must take place. Visits that occur outside the allowable visit window are considered "missed."

Table 6.1: Target days and allowable, target visit windows

Visit Number	Visit Type *(optional)*	Window periods Start date	End date	Target Day	Upper Target Window (+)	Upper Allowable Window (+)
1.0	Screening	n/a	n/a	n/a	n/a	n/a
2.0	Enrollment	n/a	n/a	0	n/a	n/a
3.0	Month 3	77 (-14)	77 (-14)	91	105 (+14)	153 (+62)
4.0	Month 6	245 (-28)	259 (-14)	273	287 (+14)	336 (+63)
5.0	Month 12	337 (-28)	351 (-14)	365	379 (+14)	426 (+61)

If a participant cannot return for a follow-up visit within the visit window, recruiters will investigate the reasons for the missed visit and complete the appropriate form:

- Missed Visit Form
- Mortality Form

All participants are followed according to their appointment schedule for the duration of the project, regardless of whether they miss one or more follow-up visits.

3-month cost-effectiveness assessment. At 3 months, we will conduct an additional assessment to evaluate the cost of the intervention as implemented in practice. This assessment will include the following: (1) time-motion studies of participants and staff to evaluate the time required for an intervention session; (2) brief surveys of patients, using the patient-cost tool described above, to evaluate the cost of attending the session from the patient perspective; (3) surveys of counselors and other healthcare staff to assess costs from the healthcare perspective; and (4) direct observation of clinic flow to evaluate the efficiency and other structural opportunity costs (e.g., waiting room space, requirement for administrative staff, overhead costs) of delivering the intervention. This assessment will be followed up with an evaluation of corresponding budgets for each item. In this way, we will be able to estimate both the full costs and the primary cost drivers of alcohol-specific interventions in this population, thereby allowing us to inform "smart" approaches to alcohol reduction in similar settings.

6.3.4 Laboratory Assessments and Specimen Storage (see also: Appendix 1)

Trained phlebotomists will collect all blood samples for the study.

Laboratory assessments and specimen storage:
Baseline
- *HIV viral load*
- *CD4*
- *HBV*
- *HCV*
- *PEth*
- *Plasma for storage*

Follow-up
- *HIV viral load – every assessment*
- *CD4 - at 12 months only*
- *PEth dried blood spots for storage – every assessment*
- *Plasma for storage – every assessment*

Blood collected for HBV and HCV will be for rapid HbsAg and HCV tests**.**

Specimen storage. We will store 10mL of blood for each participant beyond the end of the study. *We will collect plasma for storage at all four study visits: baseline, 3, 6, and 12 months.* A human biological materials repository for blood samples and dried blood spots will be maintained at the UNC Vietnam Laboratory, under the direction of the PI, Dr. Vivian Go. We would like to use these specimens to test for other health-related issues that may be related to alcohol use, HIV, hepatitis, or other sexually transmitted diseases using unspecified testing methods. *A separate informed consent for specimen storage will be administered to the participant at enrollment.* Participants do not have to agree to sign the additional consent form for specimen storage to participate in this research study. De-identified blood specimens will be stored with identification numbers for 10 years after the study is completed. Participants who agree will also be asked if they consent to be contacted for future studies. Consent forms will be used to assure that participants agree to their sample to be stored as well as to be re-contacted in the future. There are no plans to share the samples with other investigators.

6.3.5 Qualitative Cohort

Qualitative interviews will be conducted in the RCT Phase among: 1) a subset of participants and 2) all counselors.

Participant Qualitative Interviews
The main objective of these qualitative interviews will be to monitor the process of change in primary outcomes over time. Additionally, the qualitative interviews will give us an opportunity to find out which aspects of the intervention may be improved, based on the client's experience.

We will conduct a longitudinal qualitative assessments in the combined intervention and brief intervention arms. At baseline, 3, 6 and 12 months, we will conduct in-depth interviews with a sub-sample of intervention participants (including PWID, female non-PWID and a male non-PWID); this sub-sample size will be 10% of participants per intervention arm in each clinic (n = 14 per intervention arm) for a total of 28 participants in the qualitative cohort.

At each interview we will ask participants about experiences with their respective alcohol reduction program, perception of alcohol drinking and ART adherence. For example, participants will be asked about their attitudes and perceptions about drinking with specific probes on perceived norms and consequences and readiness for change and perceived skills for change. Drawing on our previous work in Vietnam that indicates the central importance of family relationships and community perceptions, we will pay close attention to issues such as community gossip about those who are perceived to drink excessively, family tensions as triggers for drinking, and family concerns about alcohol's impact on participants' health. Using a mixed methods approach, data from these interviews will be triangulated with survey data to gain a more complete understanding of the processes that underlie changes in behavior.

For each given visit, the participant's qualitative assessment will always be conducted on a date **after** the quantitative assessment, in order to not bias or influence the quantitative assessment.

Counselor Quantitative Interviews
We will also interview all 4 intervention counselors (2 trained per intervention) about their experiences of delivering the interventions. We will conduct 2 qualitative interviews per counselor at the following time points:

1) After the particular counselor has completed 25 of his/her intervention sessions.
2) After the last intervention session of the study has been delivered.

The main purpose of conducting qualitative interviews with the counselors is to help inform intervention and process findings (e.g., to help explain why the intervention did or did not work).

7.0 STUDY MEASURES

7.1 Aim 1: Co-primary outcomes

7.1.1 Alcohol use.

We will use AUDIT (validated in Vietnam[11]) to diagnose hazardous drinking (AUDIT-C score ≥ 4 for men; AUDIT-C score ≥ 3 for women) at the screening visit. In addition, we will collect detailed information on alcohol use, which we will categorize as *percent days abstinent*, *drinks per drinking day*, and *number of binge drinking days* to measure changes in drinking over time, using Alcohol Timeline Followback (TLFB). This interviewer-administered assessment reconstructs a daily behavior calendar to help prompt memory recall for alcohol consumption. TLFB has demonstrated good test–retest reliability, convergent validity, and agreement with

collateral reports for daily drinking.[88-90] Specific quantities of alcohol consumed will be collected for each day of the 30-day period prior to the intake assessment and for each 30-day period from the intake assessment up to the 3-month follow-up assessment. Daily alcohol consumption will be recorded as number of standard drinks. In general, 12 ounces of beer, 4 ounces of wine, and 1.5 ounces of liquor are each equal to 1 standard drink and in Vietnam, 2 ounces of locally brewed rice wine is equal to 1 standard drink.

An alcohol biomarker will be used on 15% of the participants to validate self- reports. Specifically, PEth is detected in whole blood by a HPLC-MS assay using dried blood spots (US Drug Testing Laboratories, Des Plaines, IL, USA). PEth is specific for alcohol metabolism and slow degradation allows detection of alcohol for ~ 3 weeks. PEth levels are useful for detecting change in drinking within subject over time. PEth has a sensitivity of 88% and specificity of 88.5% with a ROC-AUC of 0.92 for detection of any recent drinking (past 21 days) among HIV-infected persons in Uganda.[91]

7.1.2 Viral load. HIV-1 RNA levels will be performed on blood samples collected at baseline, 3, 6, and 12 months by using the *in vitro* nucleic acid amplification test (COBAS® AmpliPrep/COBAS® TaqMan® HIV-1 Test), by Roche Molecular Systems, for the quantitation of Human Immunodeficiency Virus Type 1 (HIV-1) RNA in human plasma.

7.2 Aim 2: Primary outcomes

7.2.1 Costs will be measured using direct observation and time-motion studies, detailed prospective budgetary analysis, and surveys of both participants and key staff. Costs from the patient perspective will be measured at baseline and 6 weeks. Healthcare costs will be elicited prospectively in all clinics during the counselor training and again over a two-month period during which the intervention is being deployed in each clinic.

7.2.2 Health utility will be assessed using the health utility instrument EQ-5D-5L (administered at each visit) as described above. The EQ-5D-5L also contains a visual analogue scale (VAS) for generic assessment of health utility; these scores will be compared to multi-component scale scores converted into utilities using standard weights, which we anticipate being available by the time of study implementation.

7.3 Process evaluation

7.3.1 Process measures: Through the survey, we will collect process evaluation data about client's attitudes and perceptions about drinking, and perceived barriers and facilitators to changing alcohol patterns. In addition, we will conduct 5 exit interviews per month at each clinic post-session to evaluate participants' experiences with the sessions. We will create a form to record clients' perceptions and feedback about sessions. Trainers will use these records to generate ongoing skill building exercises among the counselors. We will use process assessments from Project MATCH including client session reports completed by the client,

session checklist completed by the counselor, and counselor rating scale and fidelity ratings completed by the supervisor.

7.4 Summary of key measures

AIM 1		
**Alcohol Use**		
	AUDIT[11]	Screening, baseline and all follow-up
	Time Line Follow Back Interview [89]	Baseline and all follow-up
Alcohol biomarker	PEth[91]	Baseline<br>(PEth dried blood spot storage at all visits)
Alcohol dependence and severity	MINI[92]	Baseline
Alcohol-related consequences	SIP	
Alcohol readiness to change	Readiness to change Rulers[93] (Rollnick, 1996)	Baseline and all follow-up
Coping Skills	Alcohol abstinence self-efficacy scale of DiClemente et al. (1994)	Baseline and all follow-up
**HIV**		
Adherence	ACTG[95];	Baseline and follow-up
HIV-RNA	Laboratory	Baseline and all follow-up
CD4 Cell Count	Laboratory	Baseline and 12-months
Opportunistic Infections	Self-Report and Chart Abstraction	Baseline and all follow-up
**Hepatitis B & C**	Laboratory	Baseline
**Sexual Risk Behaviors**	Composite score	Baseline and all follow-up
**IDU Risk Behaviors**	Composite score[96]	Baseline and all follow-up
**Depressive Symptoms**	PHQ-9	Baseline and all follow-up
**Patient Provider Trust**		Baseline and all follow-up
**Violence**		Baseline and all follow-up
AIM 2		
**Healthcare system costs**	Direct observation	Baseline and months 1-3
	Time-motion studies of staff	Baseline and months 1-3
	Structured staff survey	Baseline and months 1-3
	Budgetary review	Baseline and months 1-3
**Patient costs**	Direct observation	Baseline and month 3
	Time-motion studies of patients	Baseline and month 3
	Structured patient survey	Baseline and month 3
**Health utility**	EQ-5D-5L[84]	Baseline and all follow-up

8.0 DATA ANALYSES

8.1 Statistical Analyses

For Aim 1, comparisons between interventions are focused on the 12-month follow-up, as opposed to the repeated measurements since baseline, because evidence has shown that the effect of such interventions peak early and then diminish over time.[51,53,58,77] Thus, an

intervention effect measured through, for example, an ANOVA on the repeated measurements since baseline might only be due to an early effect even if there is no sustained effect at 12 months. We will use the framework of potential outcomes for assessing time-varying interaction effects of the intervention and mediators on each primary outcome at 12 months by adjusting for the time varying factors that can be intermediate confounders – this includes the primary outcome measures at each of visits 3, 6, and 12.

In secondary analyses, we will examine the trajectory of effects over time, as we state in the Additional Considerations. For this, we give preference to cubic splines than to repeated measures ANOVA, because the standard form of the latter assumes constant variance in the error across time, whereas the former does not have such restrictions.

All tests will based on a nominal 5% two sided type I error, and confidence intervals will have nominal 95% coverage. All primary analyses will compare arms by intention-to-treat. Primary outcomes are percent days abstinent and percent viral suppression. To increase robustness, significance levels will be computed using the exact distribution of the t-test based on the permutation of the treatment arm labels. Although we expect retention rates above 90%, any missing values will be addressed by multiple imputation.

For Aim 2, we will use decision analysis with Markov modeling to project the incremental cost-effectiveness of the BI and Combined Intervention arms relative to assessment only (and to each other).

Sample size determination. For abstinent days, the sample size is determined based on the desire to detect effect sizes larger than 0.5 in three month follow-up for comparing intervention vs usual care (experience from non-US populations,[54] shows that effect sizes up to .9 are possible), and the power-sample size formula for t-test (e.g., see Piantadosi, 2005).[78] For percent viral supression, the sample size is determined based on the expected target proportions as described in the respective section, and a computer program written by our statistician relating power and sample size with expected proportions for Fisher's exact test.

8.2 Qualitative Analyses

All in-depth interviews with clinic clients and clinic providers will be audio-taped, transcribed, translated, coded and computerized for analysis using Atlas.ti v.7 qualitative data analysis software. Analysis will begin during data collection so that topics for further exploration can be incorporated into ongoing fieldwork. Qualitative data analysis consists of search for patterns in data and conceptualizing ideas that help explain the presence of those patterns.[108] Analysis of textual data will involve five steps: 1) reading for content; 2) deductive and inductive coding; 3) data display to identify emerging themes; 4) data reduction; and 5) interpretation. Memos will be written for each code. Codes will be refined during the process of analysis. Responses will be compared both within and across the different groups of participants. Moreover, in-depth interviews with program participants will be examined as longitudinal sets to analyze the impact of participation in each intervention over time.

9.0 QUALITY ASSURANCE AND QUALITY CONTROL

9.1 Intervention fidelity (Quality control)

We will conduct QA/QC via process evaluation procedures with 10% of sessions from each intervention arm, evenly distributed among the different counselors.

For both the Combined and Brief Interventions, after each session, participants will be asked to rate contents of the session on the dimensions of knowledge gained, usefulness, and clarity of information given. In addition, with permission from the participants, an audiotape will be made of all counseling sessions to assess fidelity of the session to the manual. Counselor supervisors will use process evaluation surveys and audiotapes to help maintain quality and standardization of the interventions.

For each counselor, all sessions will be reviewed for manual adherence until 5 “perfect” sessions are demonstrated. Thereafter, 25% of the sessions will be randomly selected for full review.

The Motivational Interviewing Treatment Integrity (MITI) scale will be used to code the taped sessions for treatment fidelity to provide an ongoing assessment and a record of the counselor’s competence in delivering MI. During supervision by study investigators, specific feedback will be provided to the counselor on MI competencies that meet or need improvement as well as on adherence to the manualized content.[80] To rate CBT skills, we will use the YACS[75] to assess fidelity of sessions in both interventions.

10.0 SAFETY MONITORING AND ADVERSE EVENT REPORTING

10.1 Methods for Dealing with Adverse Events

During the informed consent process, at the end of each follow-up interview, and at the beginning of each intervention session study staff will remind participants to report any physical or social harm to the study staff immediately, so that participants may receive counseling and/or other assistance. A study psychiatrist and physician will be available to study participants for the duration of the study; in cases where the psychiatrist or physician are not trained or equipped to treat the participant, referrals will be made. Participants also are asked whether they have suffered any social harm or adverse impact from the study as a routine part of follow-up interviews. Information on arrest, incarceration and drug overdose will be collected as part of the baseline and follow-up questionnaires.

When an adverse event is reported to the study staff, the staff will investigate the details of the event and will reach a conclusion as to whether the occurrence of the event was related to

participation in the study. They will refer the participant to medical, psychological, or social services as deemed appropriate; if necessary, the investigators may take action to terminate the participant from the study. All adverse events and serious adverse events will be reported to the local Principal Investigator, Mr. Pham The Vu, within 24 hours orally or by email. A completed written report of the event will be sent to the Principal Investigator, Dr. Vivian Go, within 72 hours, and to the National Institute on Drug Abuse Project Officer, the Data Safety Monitoring Board, and the Institutional Review Boards within one week.

10.2 Methods for Dealing with Illegal Reportable Activities

The Centre for Preventive Medicine in Thai Nguyen has agreements with local law enforcement personnel to ensure that participants in HIV risk reduction programs are not arrested for participation in programs.

10.3 Data Safety and Monitoring Board (DSMB)

Safety monitoring will be performed by an independent Data Safety Monitoring Committee comprised of researchers from Hanoi and individuals in Thai Nguyen with expertise in issues relevant to this study.

The committee will meet initially before the trial begins or as soon thereafter as possible. It will convene again to review the study within one year of commencing enrollment and at least annually thereafter. Emergency meetings of the committee will be convened as needed.

At each meeting to review interim reports the DSM Committee will review the following in a report prepared by a statistician external to the study team:

- Expected versus actual accrual rates
- Study retention rates by total and by study arm (unblinded)
- Study outcomes, as available by total and by study arm (unblinded)
- Number of adverse events and serious adverse events by type, by total, and by study arms (unblinded)
- Any quality assurance or regulatory issues that have occurred since the previous review
- Any actions or changes with respect to the study protocol.

Blind interim analyses of the data will be conducted halfway through the follow-up period, when virtually all participants will have provided 6-month behavioral interim endpoints. Interim reports will be prepared for the DSM Committee by an external statistician and will be presented to the protocol team blinded as to study arm, labeled as “A”, “B”, “C” “D”.

Consideration will be given to stopping the study under each of the following scenarios.

- Increases in the overall number of drug use and risk behaviors for all study participants, as compared to baseline levels, that are statistically significant at the $p<0.001$ level.
- Differences in study outcomes between the control arm (receiving neither intervention) and any of the three study arms that are statistically significant at the $p<0.001$ level.
- Differences in the number of serious adverse events (calculated by collapsing all SAEs) between the control arm (receiving neither intervention) and any of the three study arms

that are statistically significant at the $p < 0.001$ level. SAEs include mortality, suicide, accidents and violence.

When considering whether to stop the study, the overall risks and benefits experience as of the interim analysis date will be weighted by the protocol team. The recommendation of the DSM Committee and opinion of the study sponsor will be given heavy weight.

The DSM Committee will review the verbal autopsy data in aggregate from the Verbal Autopsy Study, once all of the data is collected. The DSM Committee will review the data to assess the possibility of potential harms to persons still living that may be associated with the information uncovered in the verbal autopsy. Additionally, the DSM Committee will review the data to determine whether or not any information uncovered in the verbal autopsy indicates unexpected death rates, unexpected causes of death, or any other unexpected situations that should be reported to the appropriate healthcare providers (e.g., ART providers) or authorities. Based on the results, the DSM Committee will advise us on how to proceed, and we will inform the relevant healthcare providers or authorities of any unexpected situations based on their recommendations.

We do not anticipate the DSM committee will recommend halting any focus group discussions for the sub-study on community inequality and stigma. However, if any complaints are received or participants leave or refuse to answer, a note will be made by the facilitator and reviewed and discontinued as necessary. We do not believe that the community-level questions about income, inequality and power would cause sufficient discomfort for this to happen.

Adverse events collected in reports by the study staff include not only physical harms such as mortality, suicide, accidents (traffic and other) and violence, but also social harms, such as arrest, incarceration, harassment by the police, being expelled from school or work, and instances of discrimination. Serious adverse events are based on the US Federal Drug Administration definition and are defined as events that are life-threatening or result in death, hospitalization or prolongation of existing hospitalization, a persistent or significant disability, or a congenital abnormality or birth defect. Other injuries or medical events may be considered serious adverse events, if a physician determines these events may jeopardize the participant and may require medical or surgical intervention to prevent one of the above outcomes. In our study, drug abuse or dependency alone in the absence of other adverse events will not be considered an adverse event; however patterns of drug use behavior will be analyzed. To our knowledge, no applicable laws in Vietnam exist for reporting suicide, physical abuse, sexual abuse, or intentional HIV transmission.

Adverse events and serious adverse events reported to study staff will be reported to the local PI, Mr. Pham The Vu within 24 hours orally or by email, and a completed written report of the event will be sent to the PI, Dr. Vivian Go, within 24 hours of study staff finding out about the event. A copy of the written report will also be sent to the NIDA project officer within 72 hours of the study finding out about the event, and to the DSM Committee and IRBs within one week.

Reports of the DSM Committee meetings will be provided to the Thai Nguyen Centre for Preventive Medicine IRB and the UNC IRB within 10 days.

11.0 HUMAN SUBJECTS CONSIDERATIONS

11.1 Ethical Review

This protocol, all informed consent forms, and study materials that are used directly with the participant (e.g., recruitment flyers, questionnaires, interview guides) will be reviewed and approved by the UNC IRB and the local IRB at the Thai Nguyen Center for Preventive Medicine (CPM).

The Thai Nguyen IRB is registered with the OHRP and has been granted Federal-wide Assurance (IRB00004109; FWA00007138).

11.2 Informed Consent

Informed consent will be obtained from each study participant for Screening (at the Baseline visit) prior to conducting the screening survey of the baseline visit.

After the screening survey:

- If the participant is determined *eligible* for the RCT, then the Enrollment and Stored Specimen consents will be obtained prior to conducting the remaining baseline procedures (for enrolled participants: baseline questionnaire, TLFB and baseline blood draw).
- If the participant is determined *ineligible* for the RCT, then the baseline questionnaire will be conducted only.

Our research staff has prior experience obtaining informed consent from HIV-infected persons for clinical trials within this cultural context. The informed consent procedures have been designed to maximize understanding of potential risks. All consent forms will be translated into Vietnamese and translators will sign corresponding verification of translation forms.

Consent forms will be read aloud to participants by study interviewers. After reading the consent forms and prior to seeing a signature, interviewers will ask participants to summarize the study and explain the reasons why they want to participate. At this point, any misunderstandings regarding procedures, risks, or benefits can be clarified. If there are cultural, literacy, or political reasons why a signature is not appropriate, individuals will be allowed to mark the consent form with an “X.” Informed consent will be obtained when data are collected including in-depth interviews, the screening, and baseline assessment.

If the participant is not literate, a literate witness who is **not** involved with recruitment must be present during the entire informed consent discussion. During follow-up interviews participants will be reminded about the consent form that they signed initially and any misunderstandings will

be clarified. Information sheets will be given to participants at each intervention session that include information about the research study.

11.3 Risks/Benefits

11.3.1 Description of Risks and Measures to Minimize Risks

Participants completing the baseline and follow-up assessments may have a small risk of psychological distress posed by study questions concerning alcohol use, HIV risk and sexual and drug using behaviors. Participants may find answering questions about these issues upsetting; thus, these questions will be asked in a sensitive manner. If a participant becomes emotionally upset during the interview, the research staff will be trained on how to handle these situations, and the local principal investigator, Mr. Pham The Vu, will be available to speak with participants if needed.

There are minimal physical risks to giving blood, such as a slight pain when the needle is inserted and possible bruising. To reduce the possibility of pain and bruising, blood draws will be taken by trained phlebotomists, and participants will be asked to rest and drink water following the blood draw. A psychiatrist will be on call should a counselor feel that a participant is in danger of harming himself or others during the course of a study visit.

Potential exposure to police authorities during the study may place PWIDs in a situation where they receive referral to a drug treatment program. However with commitment from both national and local officials on protecting confidentiality and careful study measures to assure confidentiality, we anticipate few challenges and no problems with local authorities. Furthermore, we have established ties to this community and have a working relationship with the local People's Committees and police authorities.

Because HIV-status is an inclusion criteria for participation in our study, we will take steps to maintain the strict confidentiality on all participants' HIV status.

11.3.2 Description of Potential Benefits

Participants in the intervention arm may benefit from learning about harmful effects of alcohol use and ways to reduce their alcohol use.

11.3.3 Compensation

Participants will be paid the 100,000 Vietnamese Dong [US $5.00] for each completed interview for the time they may have lost from work. All participants will also receive reimbursement of travel costs to compensate for transportation to and from the research facility.

11.3.4 Confidentiality Assurances

Certificate of Confidentiality/Letters of Commitment
In Vietnam, there is no certificate of confidentiality. However, letters of commitment from authorities have been obtained that assure no breaches in confidentiality.

Data Security
All participants will receive an identification number (PID), and this number will be used for all interviews, laboratory specimens, and participant study documents. No other information that would disclose the participant's identity will be found on any interview. Data will be kept without serostatus identification in locked cabinets at the Center for Preventive Medicine in Thai Nguyen. Only the consent form, tracker form, and tracker computer will link the participant's name to the identification number. To maintain contact with participants, we will use a computerized tracking system as described in the Study Procedures section. In addition, interviewers and office staff will sign a confidentiality pledge prior to having contact with participants.

We will take steps to maintain strict confidentiality of information on participants' HIV status.

In this study, procedures in place to protect subjects against risk include use of highly trained study team members. The interviewers and other study team members are highly trained in procedures for administering the sessions in a sensitive manner and in ensuring confidentiality of the participants.

All UNC-CH investigators and staff are required to successfully complete the online human subjects research ethics training program through the Collaborative Institutional Training Initiative (CITI) as well as GCP training, and they will be required to successfully complete a refresher CITI course for human subjects research every 5 years. All project staff will sign a pledge of confidentiality.

All information that the participants provide to us will be kept confidential. Information that the participants give us in this study will be kept private by the study researchers.

Staff who are involved in a breach of confidentiality will be required to meet with the Project Director to discuss the breach, why it is unacceptable, and corrective actions. That staff will not be allowed to continue working with the project until he/she completes additional research ethics training as assigned by the PI. Upon completion of the additional training, the staff will be required to sign a new pledge of confidentiality. If the same staff is involved in another breach of confidentiality, he/she will be released from working with the study.

Who Will Have Access to the Data
To ensure confidentiality, electronic data will be saved on password-protected on secure computers. Access to security passwords will be given only to the PI, Project Director, Data

Management Manager, and Site PI, Mr. Pham The Vu, at the Centre for Preventive Medicine. Personal identifiers will not be stored in the data set, and all computers will be protected by anti-virus software. Raw data files will be destroyed in the field setting by shredding one year after electronic coding. Tapes of the qualitative interviews and focus group discussions will be destroyed within one year of being transcribed electronically by crushing and cutting the tapes. Blood samples will be incinerated after 10 years of storage in lab facilities.

Additionally, all study staff undergo a training with a medical professional to recognize signs of suicidal behavior or ideation and other psychological disturbances and appropriate procedures to follow in these cases, and a psychiatrist will be on call should a counselor/facilitator feel that a participant is in danger of harming himself or others during the course of the sessions for any reason. The psychiatrist on call is:

Dr. Tran Thi Dinh
Head of Mental Health Department
Thai Nguyen Mental Health Hospital
Tel: 844 975 341 476

11.3.5 Collaborative Agreements

The study will be conducted in collaboration with the Thai Nguyen Center for Preventive Medicine (CPM), based on Memorandums of Understanding between UNC and CPM. The local IRB reviews the research plan and consent forms. The approval letter will be forwarded to the UNC IRB when it is available.

12.0 STUDY TIMELINE

**Activity**	**Year 1**				**Year 2**				**Year 3**				**Year 4**				**Year 5**			
	1	2	3	4	1	2	3	4	1	2	3	4	1	2	3	4	1	2	3	4
Planning & obtaining approvals																				
Cultural adaptation of interventions																				
Developing protocols & translating manuals																				
Staff recruitment & training																				
Initial costing																				
Screening & Enrollment																				
Baseline assessment																				
Randomization																				
Intervention																				
Follow-up assessments																				
Assessment of costs																				
Qualitative cohort interviews																				
Data cleaning & analysis, including cost-effectiveness model																				
Dissemination																				

APPENDIX 1.

Table 1: Schedule of procedures and evaluation for participants

	Baseline	Enrollment	3mo	6mo	12mo
Administrative, regulatory, and behavioral assessment procedures					
Baseline informed consent	X				
Enrollment informed consent		X			
Stored specimen informed consent	X	X			
Locator information (among enrolled only)	X		X	X	X
Baseline questionnaire	X				
Follow-up questionnaire			X	X	X
TLFB	X		X	X	X
Qualitative assessment	X		X	X	X
Cost-effectiveness questionnaire			X		
Clinical Procedures					
Retrieve physical examination data from ART clinic health records to grade AIDS clinical stage	X		X	X	X
Laboratory procedures					
HIV viral load	X		X	X	X
CD4 test	X				X
HBV HBsAg rapid test	X				
HCV rapid test	X				
PEth dried blood spot collection	X		X	X	X
PEth testing (15% of enrolled)	X				
Plasma storage	X		X	X	X

APPENDIX 2.

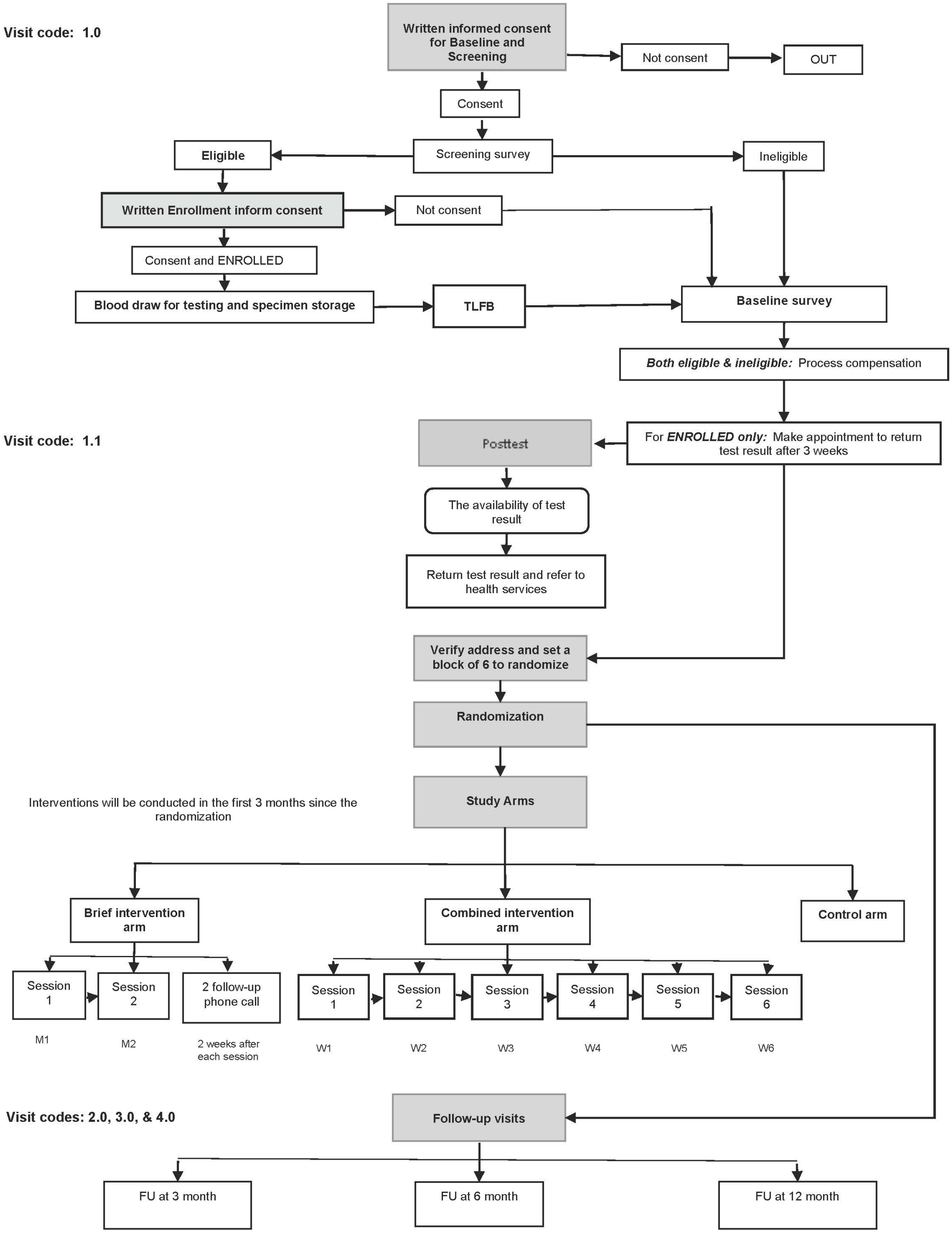
Visit code: 1.0
Written informed consent for Baseline and Screening
Not consent
OUT
Consent
Eligible
Screening survey
Ineligible
Written Enrollment inform consent
Not consent
Consent and ENROLLED
Blood draw for testing and specimen storage
TLFB
Baseline survey
Both eligible & ineligible: Process compensation
Visit code: 1.1
Posttest
For ENROLLED only: Make appointment to return test result after 3 weeks
The availability of test result
Return test result and refer to health services
Verify address and set a block of 6 to randomize
Randomization
Interventions will be conducted in the first 3 months since the randomization
Study Arms
Brief intervention arm
Combined intervention arm
Control arm
Session 1
Session 2
2 follow-up phone call
Session 1
Session 2
Session 3
Session 4
Session 5
Session 6
M1
M2
2 weeks after each session
W1
W2
W3
W4
W5
W6
Visit codes: 2.0, 3.0, & 4.0
Follow-up visits
FU at 3 month
FU at 6 month
FU at 12 month

Summary of Changes to the REDART Protocol

Protocol Version Number / Date	IRB Approval Date	Summary of Changes from Previous Version
Version 0.6 / 12 Feb 2016	14 Mar 2016	Original protocol
Version 1.0 / 21 Mar 2016	14 Dec 2016	Revised language to make details of study clearer or provide further explanation about background (no changes to meaning of content), including:<br>- Section 5.1 – Added more background on the Combined Intervention<br>- Section 5.2 – Added more background on the Brief Intervention<br>- Section 6.3.1 – Added summary of Section 6.3.1 procedures<br>- Section 6.3.2 – Updated size of randomization block from 6 to 3 participants.
Version 2.0 / 30 Nov 2016 (Final Protocol)	14 Dec 2016	Section 6.3.1 (pages 17-18) – Added “Procedures for decreasing risks from alcohol withdrawal”<br><br>Section 10.3 (page 29) – Updated timeframe for reporting adverse events and serious adverse events to the PI (24 hours) and to the NIDA project officer (72 hours)

Study Title: Reducing hazardous alcohol use & HIV viral load: an RCT in ART clinics in Vietnam (REDART)
PI: Vivian F. Go, PhD

STATISTICAL ANALYSIS PLAN

1.0 STUDY AIMS AND HYPOTHESES

Aim 1: To conduct a three-arm randomized controlled trial among hazardous and heavy drinking ART clinic patients with HIV in Vietnam comparing the effects of two evidence-based, manually guided, individually delivered interventions: Brief Intervention (BI) and Combined Intervention (CoI) with an assessment-only standard of care (SOC) control.

Aim 2: To measure the incremental cost-effectiveness of BI and CoI as compared to current counseling services offered in ART clinics in Vietnam. HIV-infected patients will be screened and evaluated for hazardous drinking behavior prior to enrollment and block randomized to either BI, CoI, or SOC. They will be interviewed at baseline, 3, 6, and 12 months after enrollment.

1.1 Hypotheses for Aim 1

1a. BI and CoI each will be more effective than an SOC arm on percent days alcohol abstinent and percent virally suppressed at 12-month assessment [**Aim 1 primary outcomes**];
1b. BI will be equivalently effective to COI on percent days alcohol abstinent [primary outcome];
1c. The effect of CoI and BI interventions on abstinence and viral suppression will be mediated by alcohol readiness to change and/or skill acquisition [**Aim 1 secondary outcomes**].
1d. CoI will be more effective than BI on abstinence and viral suppression separately among participants with more severe alcohol use, injection drug users and participants with depressive symptoms [effect modifiers].

1.2 Hypotheses for Aim 2

2a. Alcohol reduction interventions (BI and CoI) will be highly cost-effective compared to SOC, using a willingness-to-pay threshold of Vietnam's gross domestic product (GDP) per disability-adjusted life year (DALY) averted [**Aim 2 primary outcome**].
2b. BI will be highly cost-effective relative to CoI [**Aim 2 primary outcome**].

2.0 STATISTICAL ANALYSES AND POWER

2.1 Effectiveness Analysis (Aim 1). All tests will be based on a nominal 5% two-sided type I error, and confidence intervals will have nominal 95% coverage. All primary analyses will compare arms by intention-to-treat.

2.1.1 Hypothesis 1a. Combined Intervention (CoI) and Brief Intervention (BI) each will be more effective than the SOC arm on percent days alcohol abstinent and percent virally suppressed at the 12-month assessment [primary outcomes]

Percent days alcohol abstinent:
Analysis for abstinent days. We will assess this hypothesis by first calculating for each participant *i* the difference, say D_i , in percent abstinent days reported at the 12 month follow-up minus the one reported at baseline. Then we will estimate the average difference $E(D_i \mid Ti{=}BI)$ among participants *i* in treatment arm T_i =BI and compare it to the estimated average difference $E(D_i \mid Ti{=}SOC)$ among participants in treatment arm T_i =SOC, using a t-test. To increase robustness, significance levels will be computed using the exact distribution of the t-test based on the permutation of the treatment arm labels. We will do the same to compare between the CoI arm and SOC arm.

Power for abstinent days. Existing evidence from non-US populations[1] shows that BI can have an effect size up to 0.90 on D_i when compared to usual care at a three month follow-up; moreover, there is no evidence that BI differs from CoI. With 147 planned participants in each treatment arm, and

allowing for 10% missing values at the 12 month follow-up, the above t-test will have 80% power to detect an effect size of 50% or larger between BI and SOC (and between CoI and SOC).

Percent viral suppression:
Analysis for percent viral suppression. We will first estimate the probability that participants i have viral suppression, indicated by, $Y_{i,12}$ =1, at 12 months in each of the three intervention arms. Denote these three probabilities by pr($Y_{i,12}$ =1 | T_i =t), for t=BI, CoI and SOC arm respectively. Because for each participant i we will also have measured the viral suppression status at baseline, say $Y_{i,0}$ =1, will have estimate pr($Y_{i,12}$ =1 | T_i =t) by accounting for the baseline $Y_{i,0}$ =1 using the non-parametric maximum likelihood estimator (MLE) which takes the form:

$$\widehat{pr}(Y_{i,12} = 1 \mid T_i = t) = \sum_{y=0,1} \widehat{pr}\,(Y_{i,12} = 1 \mid T_i = t, Y_{i,0} = y) * \widehat{pr}\,(Y_{0,i} = y) \qquad \text{(expr. 1)}$$

where $\widehat{pr}\,(Y_{0,i} = y)$, by randomization, is estimated from all arms combined. We will then compare BI versus SOC by the odds ratio (OR) of the probabilities pr($Y_{i,12}$ =1 | T_i =BI) versus pr($Y_{i,12}$ =1 | T_i =SOC) using the permutation distribution of the treatment labels; the same method will be used to compare CoI versus SOC.

Power for percent viral suppression. Based on literature review,[2,3] we expect that approximately 70% of participants will have viral suppression ($Y_{i,0}$ =1) at baseline, that most of those participants will remain virally suppressed at the 12 month follow-up in each of the three arms; that an additional 2% (total 72%) will become virally suppressed at the SOC arm; and that an additional 15% (total 85%) can become virally suppressed at 12 months in each of the CoI and BI arms. Given this, the exact power of the test statistic OR based on $\widehat{pr}$($Y_{i,12}$ =1 | T_i =t) for t=BI (or CoI) versus t=SOC is the same as the power of the fisher exact test for comparing viral suppression at 12 months between BI and SOC arm among those who will not be virally suppressed at baseline, i.e. comparing $\widehat{pr}\,(Y_{i,12} = 1 \mid T_i = t, Y_{i,0} = 0)$ between arms t. Allowing for 10% missing values at the end of follow-up, the study has more than 80% power to detect an 80% viral suppression at 12 months for all participants at BI (up from 70% at baseline) compared to the 72% (70%+2%) for all participants at the SOC arm. This effect size is feasible as it is smaller than the one above (72% versus 85%) suggested by the literature.

2.1.2 Hypothesis 1b. BI will be equivalently effective to CoI on percent days alcohol abstinent.

Analysis. We will estimate the 95% CI for the effect size e.s.=$|E(D_i \mid T_i\text{=BI})-E(D_i \mid T_i\text{=SOC})|/\sigma$ (where σ is the pooled standard deviation of D_i).

Power. We will declare that BI and CoI are equivalent if the estimate of the effect size e.s. is within a tolerance window. Based on Piantodosi,[4] a 5% type-I error test has 80% power to declare that the estimated effect size is within a window of [0± 0.35] (with 147 participants per arm and a 10% missingness rate) if BI and CoI are truly equivalent.

2.1.3 Hypothesis 1c. The effect of CoI and BI interventions on abstinence and viral load will be mediated by alcohol readiness to change and/or skill acquisition [secondary outcomes].

Analysis for mediation of effect on abstinence. Because both the mediators and abstinence are longitudinal, the measurement of abstinence at a visit can be simultaneously affected by past measurements of the mediators as well as a predictor of future measurements of the mediators. For this reason, to assess this hypothesis we will use the framework of potential outcomes for assessing time-varying interaction effects of the intervention and mediators on abstinence at 12 months by adjusting for the time varying factors that can be intermediate confounders – this includes the primary outcome measures at each of visits 3, 6, and 12. This approach, too lengthy to be detailed here, is now considered essential for addressing analogous problems with longitudinal mediators.[5,6] Our team statistician is an expert in the area of time-varying treatments, with both established and continuing significant contributions in the field.[7,8]

Analysis for mediation of effect on viral load. This will also be based on the framework of potential outcomes for assessing time-varying interaction effects between the intervention and mediators as referenced as above.

2.1.4 Hypothesis 1d. CoI will be more effective than BI on abstinence and viral load for females, injection drug users and participants with depressive symptoms [effect modifiers, secondary analyses].

Potential effect modification on abstinence will be assessed using a linear regression of D_i (see Hypothesis 1a) on the intervention (CoI vs BI) interacting with the potential effect modifier, first in separate analyses for each modifier, and then in an analysis with as many modifiers together as possible. The assessment of effect modification for a factor will be based on assessing the importance of the corresponding interaction term using robust standard errors. Potential effect modifiers X_i on viral load suppression will be assessed using a logistic regression for $pr\left(Y_{i,12} = 1 \mid X, T_i, Y_{i,0}\right)$ (see Hypothesis 1a) with the intervention (T_i= CoI vs BI) interacting with the potential effect modifier X_i , as described for abstinence.

2.1.5 Additional considerations. For Hypothesis 1a, secondary analyses will model the trajectories of the primary outcomes across visits with cubic splines to assess dependence of effects over time. Although we expect retention rates above 90%, any missing values will be addressed by multiple imputation.

2.2 Cost-Effectiveness Analysis (Aim 2)

We will use decision analysis with Markov modeling to project the incremental cost-effectiveness of the BI and CoI arms relative to SOC (and to each other). In this model, we will use study data to develop a simulated cohort of HIV-infected individuals in ART care, assigning each individual to HIV disease states according to CD4 count, alcohol use, and HIV RNA viral load (suppressed or not suppressed), with health outcomes (e.g., opportunistic infections, HIV transmission events) conditional on the disease state in which an individual resides. This cohort will be followed for HIV disease progression over time using a Markov process, with the probability of each subsequent transition reflecting the individual's alcohol use and viral suppression status[9] at any given time. Using the primary effectiveness data from Aim 1, we will simulate HIV disease progression under each of the three study interventions (CoI, BI, or SOC). Each health state will be assigned a health utility score (using study data and comparison to existing literature) and a cost (using study data to estimate the cost of ART and alcohol intervention, literature to estimate the probability of complications and the cost of treating them), and the progression of the simulated cohort through these states will be used to project the total cost and health utility associated with each intervention. Thus, we will be able to analyze the benefits of alcohol-related interventions (e.g., reduction in viral load leading to reduced HIV disease progression, improved quality of life with less AUD) as those benefits accrue over patients' entire lifetimes.

Using this model, we will estimate the incremental cost-effectiveness ratio of each study arm (ICER, defined as $[C_i – C_a]/[E_i – E_a]$, with C_i and C_b being the respective costs of the intervention [i] and SOC [a], and E_i and E_b the corresponding effectiveness). Our primary effectiveness measure will be number of DALYs averted, using standard disability weights from the Global Burden of Disease study.[10] In our primary analysis, we will adopt a societal perspective and lifetime time horizon, as recommended by international guidelines.[11] However, we will also conduct alternative analyses from the healthcare perspective and using a time horizon of one year (i.e., assuming that all impacts of the intervention are felt only within the first year). In the primary analysis, we will assume that the alcohol-reduction interventions avert DALYs through two potential mechanisms, each informed by study data through the co-primary outcomes in Aim 1: (a) improved ART adherence and thus viral suppression, leading to slower HIV disease progression and thus longer and higher-quality life; and (b) direct reduction in alcohol use, thereby improving health utility (e.g., fewer depressive symptoms, assuming that participants may rate health states with less alcohol use as higher-utility states). In secondary analyses, we will explore the possible extent to which reductions in HIV transmission (i.e., fewer risky sexual or injection behaviors) and improvements in liver-related outcomes (i.e., slower progression to cirrhosis and/or liver cancer due to less alcohol intake) may also contribute to the effectiveness of the intervention. Although we will lack

sufficient power to directly project estimate these longer-term effects, our model will provide policymakers with vital information as they consider whether alcohol-related interventions are a cost-effective use of limited resources for people living with HIV/AIDS.

We will perform extensive sensitivity and uncertainty analysis in association with this model. Sensitivity analyses will include one-way sensitivity analysis on all model parameters as well as multi-way analyses on those parameters found to have greatest influence on incremental cost-effectiveness. We will conduct subgroup uncertainty analyses, where we consider cohorts specifically comprised of injecting drug users, individuals with depressive symptoms, only women, and only men – each reflecting key subgroups in our analysis of Aim 1. To estimate the uncertainty in our estimates of incremental cost-effectiveness, we will conduct a probabilistic uncertainty analysis, using estimates of variability from Aim 1 (i.e., confidence intervals around the primary effectiveness measures) and the literature (for parameters, e.g., rate of HIV disease progression according to viral load, not directly estimated from the study) to simultaneously vary all model parameters over thousands of simulations. We will use these simulations to construct 95% uncertainty ranges over each of our modeled cost-effectiveness estimates, and to construct cost-effectiveness acceptability curves describing the probability of cost-effectiveness across a range of willingness-to-pay thresholds. Thus, we will not only evaluate cost-effectiveness, but we will provide decision-makers with the information they need regarding uncertainty in cost-effectiveness estimates and what that cost-effectiveness might be in settings with different epidemiological or sociocultural characteristics.

References

1. Papas RK, Sidle JE, Gakinya BN, et al. Treatment outcomes of a stage 1 cognitive-behavioral trial to reduce alcohol use among human immunodeficiency virus-infected out-patients in western Kenya. *Addiction.* 2011;106(12):2156-2166.
2. Jordan MR, La H, Nguyen HD, et al. Correlates of HIV-1 viral suppression in a cohort of HIV-positive drug users receiving antiretroviral therapy in Hanoi, Vietnam. *Int J STD AIDS.* 2009;20(6):418-422.
3. Trinh TT, Montague BT, Flanigan TP, Gerard HM. HIV Suppression among Patients on Treatment in Vietnam: A Review of HIV Viral Load Testing in a Public Urban Clinic in Ho Chi Minh City. *AIDS Res Treat.* 2011;2011:230953.
4. Piantadosi S. *Clinical Trials: A Methodologic Perspective.* New York: Wiley; 1997.
5. Robins J. A graphical approach to the identification and estimation of causal parameters in mortality studies with sustained exposure periods. *J Chronic Dis.* 1987;40 Suppl 2:139S-161S.
6. Robins JM, Hernan MA, Brumback B. Marginal structural models and causal inference in epidemiology. *Epidemiology.* 2000;11(5):550-560.
7. Frangakis CE, Rubin DB. Principal stratification in causal inference. *Biometrics.* 2002;58(1):21-29.
8. Shinohara RT, Narayan AK, Hong K, et al. Estimating parsimonious models of longitudinal causal effects using regressions on propensity scores. *Stat Med.* 2013.
9. Mellors JW, Munoz A, Giorgi JV, et al. Plasma viral load and CD4+ lymphocytes as prognostic markers of HIV-1 infection. *Ann Intern Med.* 1997;126(12):946-954.
10. Salomon JA, Vos T, Hogan DR, et al. Common values in assessing health outcomes from disease and injury: disability weights measurement study for the Global Burden of Disease Study 2010. *Lancet.* 2012;380(9859):2129-2143.
11. Tan-Torres Edejer T. *Making Choices in Health: WHO Guide to Cost-Effectiveness Analysis.* Geneva: World Health Organization;2003.
