# Supplementary material for: Effect of 2 Integrated Interventions on Alcohol Abstinence and Viral Suppression Among Vietnamese Adults With Hazardous Alcohol Use and HIV: A Randomized Clinical Trial
Source: JAMA Netw Open. 2020 Sep 18;3(9):e2017115. doi: 10.1001/jamanetworkopen.2020.17115 (PMC7501538; doi:10.1001/jamanetworkopen.2020.17115)
Supplement: Supplement 2. — eTable 1. Number of Participants Enrolled in the Study From Each ART Clinic, Thai Nguyen Province, Vietnam eTable 2. Descriptions of Each Alcohol Reduction Intervention Evaluated eTable 3. Cohen d for Difference Among Groups for Mean Percentage of Days Abstinent From Alcohol in the Last 30 Days eTable 4. Difference Among Groups for Percentage Viral Suppression in the Last 30 Days eTable 5. Observed Effect Size vs Planned Power for Primary Outcomes eTable 6. Mean Number of Drinks per Drinking Day in the Last 30 Days, by Groups and Visits eTable 7. Cohen d for Difference Among Groups for Mean Number of Drinks per Drinking Day in the last 30 Days eTable 8. Mean Number of Heavy Drinking Days in the Last 30 Days, by Groups and Visits eTable 9. Cohen d for Difference Among Groups for Mean Number of Heavy Drinking Days in the last 30 Days eTable 10. Self-reported Alcohol Abstinence in the Last 3 weeks and Phosphatidylethanol Levels Less Than 8 ng/mL [file jamanetwopen-e2017115-s002.pdf]

Supplementary Online Content

Go VF, Hutton HE, Ha TV, et al. Effect of 2 integrated interventions on alcohol abstinence and viral suppression among Vietnamese adults with hazardous alcohol use and HIV: a randomized clinical trial. *JAMA Netw Open*. 2020;3(9):e2017115. doi:10.1001/jamanetworkopen.2020.17115

eTable 1. Number of Participants Enrolled in the Study From Each ART Clinic, Thai Nguyen Province, Vietnam
eTable 2. Descriptions of Each Alcohol Reduction Intervention Evaluated
eTable 3. Cohen *d* for Difference Among Groups for Mean Percentage of Days Abstinent From Alcohol in the Last 30 Days
eTable 4. Difference Among Groups for Percentage Viral Suppression in the Last 30 Days
eTable 5. Observed Effect Size vs Planned Power for Primary Outcomes
eTable 6. Mean Number of Drinks per Drinking Day in the Last 30 Days, by Groups and Visits
eTable 7. Cohen *d* for Difference Among Groups for Mean Number of Drinks per Drinking Day in the last 30 Days
eTable 8. Mean Number of Heavy Drinking Days in the Last 30 Days, by Groups and Visits
eTable 9. Cohen *d* for Difference Among Groups for Mean Number of Heavy Drinking Days in the last 30 Days
eTable 10. Self-reported Alcohol Abstinence in the Last 3 weeks and Phosphatidylethanol Levels Less Than 8 ng/mL

This supplementary material has been provided by the authors to give readers additional information about their work.

© 2020 Go VF et al. *JAMA Network Open.*

eTable 1. Number of Participants Enrolled in the Study From Each ART Clinic, Thai Nguyen Province, Vietnam

**ART Clinic**	**N (%)**
Medical Center of Thai Nguyen	75 (17.1)
Medical Center of Dong Hy District	60 (13.6)
Medical Center of Dai Tu District	114 (25.9)
The A Hospital	89 (20.2)
Medical Center of Phu Binh District	46 (10.5)
Medical Center of Phu Luong District	55 (12.5)
Medical Center of Pho Yen District	1 (0.2)

Abbreviations: ART, antiretroviral therapy.

© 2020 Go VF et al. *JAMA Network Open.*

eTable 2. Descriptions of Each Alcohol Reduction Intervention Evaluated

Intervention	Components	No. sessions	Duration of each session	Additional elements
Combined Intervention (CoI)	• Personalized feedback<br>• Consideration of pros and cons<br>• Strategies for managing risky moods and situations for alcohol use<br>• Alcohol refusal skills<br>• Managing negative thoughts<br>• Engaging supportive others	6	45-60 minutes	3 optional group sessions, 60 minutes per session
Brief Intervention (BI)	• Personalized feedback<br>• Consideration of pros and cons<br>• Strategies for managing risky moods and situations for alcohol use	2	30-45 minutes	2 phone calls, 5 minutes per call

© 2020 Go VF et al. *JAMA Network Open.*

eTable 3. Cohen *d* for Difference Among Groups for Mean Percentage of Days Abstinent From Alcohol in the Last 30 Days

	**CoI vs SOC**	**BI vs SOC**	**CoI vs BI**
**Baseline**			
Pooled baseline SD: 33%			
d (SE)	-10% (12%)	1% (12%)	-11% (12%)
95% CI	(-33% to 13%)	(-22% to 23%)	(-34% to 12%)
P value	.38	.95	.35
**3 Months**			
Pooled baseline SD: 36%			
d (SE)	46% (12%)	64% (12%)	-18% (12%)
95% CI	(22% to 70%)	(40% to 88%)	(-41% to 6%)
P value	< .001	< .001	.14
**6 Months**			
Pooled baseline SD: 37%			
d (SE)	36% (12%)	44% (12%)	-8% (12%)
95% CI	(12% to 60%)	(20% to 68%)	(-31% to 15%)
P value	.003	< .001	.50
**12 Months**			
Pooled baseline SD: 36%			
d (SE)	39% (13%)	39% (13%)	0% (12%)
95% CI	(15% to 64%)	(15% to 64%)	(-24% to 24%)
P value	.002	.002	.99

Abbreviations: CoI, Combined Intervention; SOC, Standard of Care; BI, Brief Intervention; SD=pooled standard deviation; d=Cohen's d for difference; SE, standard error; CI, confidence interval.

© 2020 Go VF et al. *JAMA Network Open.*

eTable 4. Difference Among Groups for Percentage Viral Suppression in the Last 30 Days

	**CoI vs SOC**		**BI vs SOC**		**CoI vs BI**	
	**Raw**	**Standardized**	**Raw**	**Standardized**	**Raw**	**Standardized**
**Baseline**						
Difference (SE)	4% (4%)	0	8% (4%)	0	-4% (4%)	0
95% CI	(-4% to 12%)	-	(0% to 16%)	-	(-12% to 4%)	-
P value	.35		.06		.35	
**3 Months**						
Difference (SE)	-4% (4%)	-5% (4%)	4% (4%)	0% (4%)	-8% (4%)	-5% (4%)
95% CI	(-12% to 4%)	(-13% to 3%)	(-4% to 12%)	(-8% to 8%)	(-16% to 0%)	(-13% to 3%)
P value	.35	.24	.35	1.00	.06	.24
**6 Months**						
Difference (SE)	3% (4%)	2% (4%)	4% (4%)	0% (4%)	-1% (4%)	2 (4)
95% CI	(-5% to 11%)	(6% to 10%)	(4% to 12%)	(-8% to 8%)	(-9% to 7%)	(-6% to 10%)
P value	.48	.64	.35	1.00	.81	.64
**12 Months**						
Difference (SE)	6% (5%)	5% (5%)	15% (4%)	11% (4%)	-9% (4%)	-6% (4%)
95% CI	(-4% to 16%)	(-5% to 15%)	(6% to 24%)	(2% to 20%)	(-16% to -2%)	(-13% to 1%)
P value	.23	.32	.001	.01	.01	.10

Abbreviations: CoI, Combined Intervention; SOC, Standard of Care; BI, Brief Intervention; SE, standard error; CI, confidence interval.

© 2020 Go VF et al. *JAMA Network Open.*

eTable 5. Observed Effect Size vs Planned Power for Primary Outcomes

	Combined Intervention (CoI)	Brief Intervention (BI)	Standard of Care (SOC)
**Percentage of days abstinent**			
Baseline SD within arm: 33%			
12-month minus baseline	65% - 37% = 28%	65% - 41%= 24%	50% - 41% = 9%
(12-month minus baseline) / SD	28% / 33% = 85%	24% / 33% = 73 %	9% / 33% = 27%
Intervention arm – SOC arm effect sizes:			
As observed	85% - 27%= 58%	73% - 27%= 46%	
As planned in proposal to have >80% to show difference from arm SOC	>50%	>50%	
**Viral load**			
12-month minus baseline	83% - 84% = -1%	92% - 88% = 4%	77% - 80% = -3%
Intervention arm – SOC arm percents:			
As observed	-1% - (-3%) = 2%	4% - (-3%)= 7%	
As planned in proposal to have >80% to show difference from arm SOC	>8%	>8%	

Abbreviations: SD, standard deviation.

© 2020 Go VF et al. *JAMA Network Open.*

eTable 6. Mean Number of Drinks per Drinking Day in the Last 30 Days, by Groups and Visits[a]

	Overall	Combined Intervention (CoI)	Brief Intervention (BI)	Standard of Care (SOC)	*P* value
**Baseline**					
No.[b]	435	145	146	144	.80
Mean number of drinks per drinking day (SE)	4.1	4.1 (0.2)	4.0 (0.3)	4.0 (0.3)	.92
**3 Months**					
No.[b]	372	128	115	129	.04
Mean drinks per drinking day at 3 months (SE)	3.2	2.5 (0.2)	3.0 (0.3)	4.0 (0.3)	.001
**6 Months**					
No.[b]	375	126	121	128	.43
Mean drinks per drinking day at 6 months (SE)	3.2	2.4 (0.2)	2.9 (0.3)	4.1 (0.3)	<.001
**12 Months**					
No.[b]	361	125	116	120	.39
Mean drinks per drinking day at 12 months (SE)	3.5	2.9 (0.2)	3.4 (0.3)	4.2 (0.3)	.001
**Among those who attended each follow-up visit, baseline average drinks per drinking day[c]:**					
**3 Months**					
Mean drinks per drinking day at baseline (SE)	4.0	4.1 (0.3)	3.8 (0.3)	4.1 (0.3)	.69
**6 Months**					
Mean drinks per drinking day at baseline (SE)	4.0	4.1 (0.3)	3.8 (0.3)	4.0 (0.3)	.80
**12 Months**					
Mean drinks per drinking day at baseline (SE)	4.0	4.2 (0.3)	3.9 (0.3)	4.0 (0.3)	.83

Abbreviations: SE, standard error.
[a] 1 drink = 1 U.S. standard drink.
[b] Number of participants who drank in the past 30 days at the given visit.
[c] *P* > .65 for comparison across arms.

© 2020 Go VF et al. *JAMA Network Open.*

eTable 7. Cohen *d* for Difference Among Groups for Mean Number of Drinks per Drinking Day in the last 30 Days

	**CoI vs SOC**	**BI vs SOC**	**CoI vs BI**
**Baseline**			
Pooled baseline SD: 3.1			
d (SE)	4% (11%)	-1% (12%)	4% (12%)
95% CI	(-19% to 26%)	(-24% to 23%)	(-19% to 27%)
P value	0.74	0.96	0.71
**3 Months**			
Pooled baseline SD: 2.8			
d (SE)	-57% (12%)	-38% (14%)	-19% (12%)
95% CI	(-80% to -34%)	(-66% to -10%)	(-43% to 5%)
P value	< 0.001	0.007	0.13
**6 Months**			
Pooled baseline SD: 2.8			
d (SE)	-61% (12%)	-44% (14%)	-17% (12%)
95% CI	(-85% to -38%)	(-73% to -16%)	(-40% to 5%)
P value	<0.001	0.002	0.14
**12 Months**			
Pooled baseline SD: 2.8			
d (SE)	-46% (13%)	-26% (14%)	-20% (12%)
95% CI	(-71% to -21%)	(-54% to 2%)	(-43% to 3%)
P value	<0.001	0.07	0.09

Abbreviations: CoI, Combined Intervention; SOC, Standard of Care; BI, Brief Intervention; SD=pooled standard deviation; d=Cohen’s d for difference; SE, standard error; CI, confidence interval.

© 2020 Go VF et al. *JAMA Network Open.*

eTable 8. Mean Number of Heavy Drinking Days in the Last 30 Days, by Groups and Visits[a]

	**Overall**	**Combined Intervention (CoI)**	**Brief Intervention (BI)**	**Standard of Care (SOC)**	***P* value**
**Baseline**					
No. attended visit	440	147	147	146	
Mean heavy drinking days (SE)	7.3	8.0 (0.9)	6.8 (0.8)	6.9 (0.8)	.57
**3 Months**					
No. attended visit[b]	405	135	136	134	
Mean heavy drinking days at 3 months (SE)	4.6	3.1 (0.6)	3.6 (0.7)	7.2 (0.9)	.001
**6 Months**					
No. attended visit[b]	410	141	136	133	
Mean heavy drinking days at 6 months (SE)	4.1	2.0 (0.5)	3.3 (0.7)	7.1 (1.0)	<.001
**12 Months**					
No. attended visit[b]	390	135	129	126	
Mean heavy drinking days at 12 months (SE)	4.6	3.4 (0.7)	3.7 (0.7)	6.7 (1.0)	.01
**Among those who attended each follow-up visit, baseline average heavy drinking days[b]:**					
**3 Months**					
Mean heavy drinking days at baseline (SE)	7.1	7.6 (0.9)	6.7 (0.9)	7.1 (0.9)	
**6 Months**					
Mean heavy drinking days at baseline (SE)	7.1	7.9 (0.9)	6.7 (0.9)	6.8 (0.9)	
**12 Months**					
Mean heavy drinking days at baseline (SE)	7.2	8.0 (0.9)	6.7 (0.9)	6.9 (0.9)	

Abbreviations: SE, standard error.
[a] Heavy drinking day defined as having >4 drinks per day for males or >3 drinks per day for females.[32] 1 drink = 1 U.S. standard drink.
[b] P > .22 for comparison across arms.

© 2020 Go VF et al. *JAMA Network Open.*

eTable 9. Cohen *d* for Difference Among Groups for Mean Number of Heavy Drinking Days in the last 30 Days

	**CoI vs SOC**	**BI vs SOC**	**CoI vs BI**
**Baseline**			
Pooled baseline SD: 10%			
d (SE)	10% (12%)	-1% (11%)	11% (12%)
95% CI	(-13% to 34%)	(-23% to 21%)	(-12% to 34%)
P value	0.38	0.93	0.33
**3 Months**			
Pooled baseline SD: 9%			
d (SE)	-47% (13%)	-41% (13%)	-5% (10%)
95% CI	(-72% to -21%)	(-67% to -16%)	(-26% to 15%)
P value	<0.001	0.002	0.61
**6 Months**			
Pooled baseline SD: 8%			
d (SE)	-60% (13%)	-45% (14%)	-15% (10%)
95% CI	(-85% to -35%)	(-72% to -17%)	(-35% to 4%)
P value	<0.001	0.001	0.12
**12 Months**			
Pooled baseline SD: 9%			
d (SE)	-38% (13%)	-34% (13%)	-1% (12%)
95% CI	(-64% to -12%)	(-60% to -8%) 0.01	(-25% to 17%)
P value	0.01	0.01	0.70

Abbreviations: CoI, Combined Intervention; SOC, Standard of Care; BI, Brief Intervention; SD=pooled standard deviation; d=Cohen's d for difference; SE, standard error; CI, confidence interval.

© 2020 Go VF et al. *JAMA Network Open.*

eTable 10. Self-reported Alcohol Abstinence in the Last 3 weeks and Phosphatidylethanol (PEth) Levels Less Than 8 ng/mL

	**No alcohol use, self-reported last 3 weeks No. (%)**	**Any alcohol use, self-reported last 3 weeks No. (%)**	**OR (95% CI)**
**Baseline (N=431 observations with PEth)**			
PEth <8 ng/mL	4 (1%)	39 (9%)	9.8 (2.3 to 40.9)
PEth ≥8 ng/mL	4 (1%)	384 (89%)	Reference
**3 Months (N=401 observations with PEth)**			
PEth <8 ng/mL	18 (4%)	43 (11%)	5.8 (2.9 to 11.6)
PEth ≥8 ng/mL	23 (6%)	317 (79%)	Reference
**12 Months (N=389 observations with PEth)**			
PEth <8 ng/mL	23 (6%)	61 (16%)	6.4 (3.2 to 12.7)
PEth ≥8 ng/mL	17 (4%)	288 (74%)	Reference

Abbreviations: PEth, phosphatidylethanol biomarker for alcohol; OR, odds ratio; CI, confidence interval.

© 2020 Go VF et al. *JAMA Network Open.*
