# Supplementary material for: Effect of 2 Integrated Interventions on Alcohol Abstinence and Viral Suppression Among Vietnamese Adults With Hazardous Alcohol Use and HIV: A Randomized Clinical Trial
Source: JAMA Netw Open. 2020 Sep 18;3(9):e2017115. doi: 10.1001/jamanetworkopen.2020.17115 (PMC7501538; doi:10.1001/jamanetworkopen.2020.17115)
Supplement: Supplement 3. — Data Sharing Statement [file jamanetwopen-e2017115-s003.pdf]

Data Sharing Statement

Go. Effect of 2 Integrated Interventions on Alcohol Abstinence and Viral Suppression Among Vietnamese Adults With Hazardous Alcohol Use and HIV. *JAMA Netw Open*. Published September 18, 2020.
10.1001/jamanetworkopen.2020.17115

Data
Data available: Yes
Data types: Deidentified participant data
How to access data: vgo@unc.edu
When available: With publication

Supporting Documents
Document types: None

Additional Information
Who can access the data: Data will be made available to researchers whose proposed use of the data has been approved
Types of analyses: These data may be used for replication of these study results and individual participant data meta-analysis.
Mechanisms of data availability: To obtain the data, investigators must submit a proposal describing the intended use of the data. The proposals will be reviewed by an independent review committee (that does not include any of the authors).
